# Hidden among the crowd: differential DNA methylation-expression correlations in cancer occur at important oncogenic pathways

**DOI:** 10.3389/fgene.2015.00163

**Published:** 2015-05-13

**Authors:** Adrián Mosquera Orgueira

**Affiliations:** Independent ResearcherOrdes, Spain

**Keywords:** DNA methylation, gene expression, cancer

## Abstract

DNA methylation is a frequent epigenetic mechanism that participates in transcriptional repression. Variations in DNA methylation with respect to gene expression are constant, and, for unknown reasons, some genes with highly methylated promoters are sometimes overexpressed. In this study we have analyzed the expression and methylation patterns of thousands of genes in five groups of cancer and normal tissue samples in order to determine local and genome-wide differences. We observed significant changes in global methylation-expression correlation in all the neoplasms, which suggests that differential correlation events are frequent in cancer. A focused analysis in the breast cancer cohort identified 1662 genes whose correlation varies significantly between normal and cancerous breast, but whose DNA methylation and gene expression patterns do not change substantially. These genes were enriched in cancer-related pathways and repressive chromatin features across various model cell lines, such as PRC2 binding and H3K27me3 marks. Substantial changes in methylation-expression correlation indicate that these genes are subject to epigenetic remodeling, where the differential activity of other factors break the expected relationship between both variables. Our findings suggest a complex regulatory landscape where a redistribution of local and large-scale chromatin repressive domains at differentially correlated genes (DCGs) creates epigenetic hotspots that modulate cancer-specific gene expression.

## Introduction

DNA methylation is a repressive epigenetic phenomenon that occurs at CpG dinucleotides (Saxonov et al., 2006). These dinucleotides frequently form CpG-rich clusters known as CpG Islands, which are located near the promoter of 72% of the genes (Saxonov et al., 2006). The methylation status of the promoter is widely accepted to exert a repressive effect on proximal gene expression (Klipp, 2009; Han et al., 2011; Vanderkraats et al., 2013; Szulwach and Jin, 2014). However, the effect of DNA methylation on gene expression varies with respect to its location and the genotype of the individual (Bell et al., 2011; Lou et al., 2014; Wagner et al., 2014). For example, CpGs inside gene bodies are more positively correlated with gene expression than promoter-associated CpGs (Lou et al., 2014), and CpGs in the vicinity of CpG islands (known as *CpG island shores*) are enriched in tissue and cancer-specific differentially methylated regions (cDMRs) when compared with CpG islands (Doi et al., 2009).

The effect of DNA methylation on gene expression is an active field of research and various mechanisms have been described. Notably, DNA methylation is coupled to histone modifications through methyl-binding and methyl-insensitive factors (Szulwach and Jin, 2014). Histone deacetylase repressor complexes are targeted to methylated regions, whilst the MLL family of histone methyltransferases and the histone demethylase JHDM1a preferentially bind to demethylated DNA (Szulwach and Jin, 2014). Extensive research on epigenetics has been devoted to disentangle the role that DNA methylation plays in cancer (Ghavifekr et al., 2014). This has led to terms such as “*hypermethylated*” and “*hypomethylated*” referring to genes whose methylation changes significantly between tumor and normal tissues. However, there is evidence indicating that genes with heavily methylated promoters can be intensely expressed (Guillaumet-Adkins et al., 2014), whilst partially methylated genomic regions and long-range hypomethylated domains contain silenced genes in various cancer types (Berman et al., 2011; Hansen et al., 2011; Hon et al., 2012). DNA methylation stability is known to be lost in cancer (Hansen et al., 2011), but we found no studies addressing the potential differences and functional implications of differential gene-expression correlation with DNA methylation between tumor and normal tissues.

**Figure 1:**
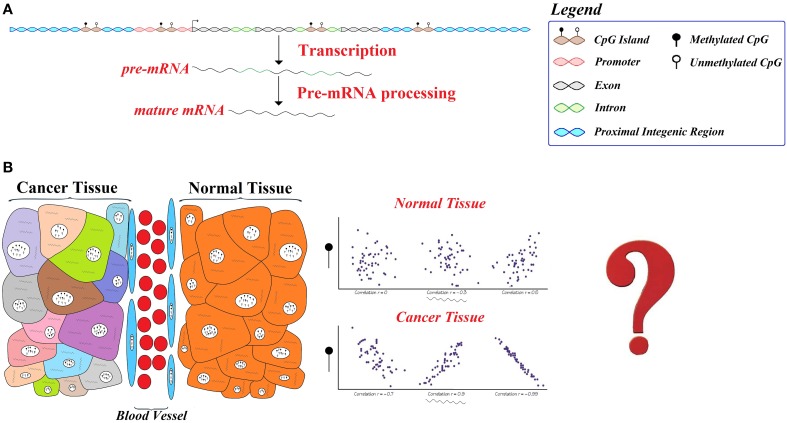
**Schematic representation of the reasoning behind this study. (A)** DNA methylation is an epigenetic modification known to modulate gene expression. **(B)** In this study, we decided to identify significant differences between tumor and normal tissues in their gene expression correlation with DNA methylation; as well as the epigenetic factors that can explain this behavior.

In this research, we studied the global and gene-by-gene patterns of differential gene expression-methylation in a set of publically available tumor-normal datasets (Figure 1). This revealed interesting differential patterns of correlation in cancer. In order to further explore the importance of these differences, we calculated gene-level statistics in normal and cancerous breast, which detected thousands of breast *Differentially Correlated Genes* (hereafter known as DCGs). Functional annotation and comparison of this list of genes with pathways, ontology and transcription factor (TF) binding regions reported by the Encode Project Consortium (ENCODE Project Consortium, 2012) provided evidence for consistent enrichments in cellular processes and chromatin modifications. The repressive effect of DNA methylation on transcription is a well described phenomenon (Saxonov et al., 2006), and we try to address the nature and implications of these findings on our understanding of epigenetics and cancer biology.

## Methods

**Figure 2:**
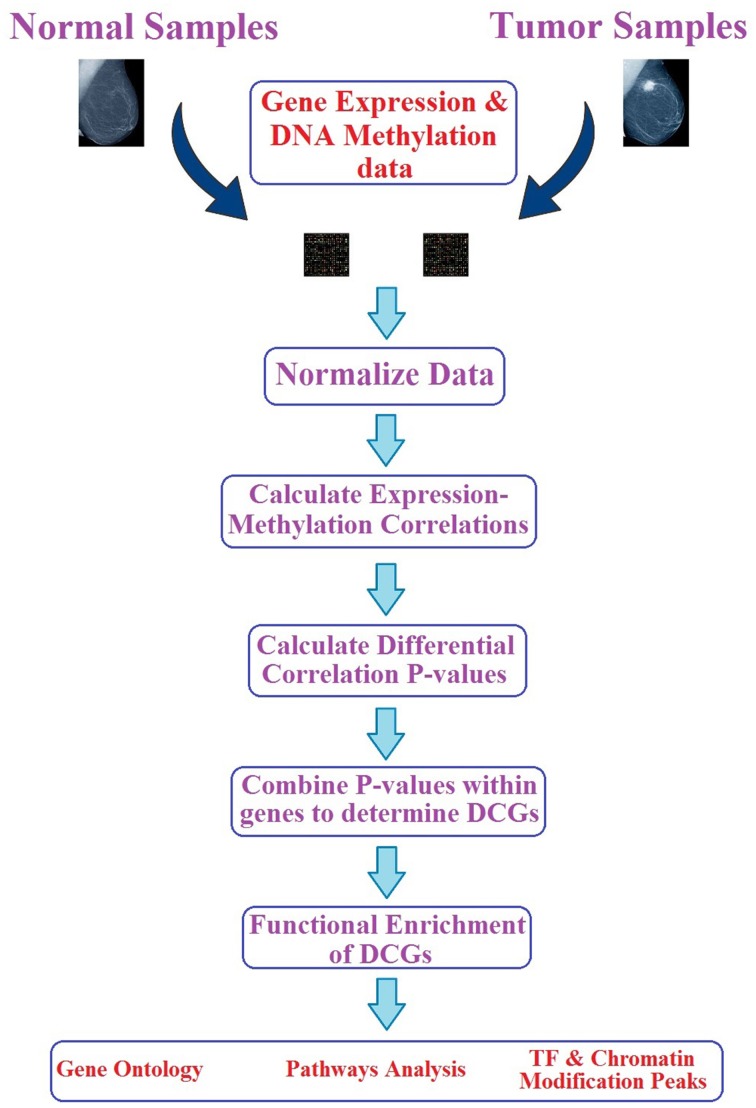
**Pipeline specifying the most important steps of the methodology used to discover DCGs and downstream analysis**.

A pipeline of the methodology used in this section can be consulted in Figure 2.

### Data source and pre-processing

Five tumor-normal datasets from the *Gene Expression Ommnibus* (GEO) (Barrett et al., 2013) with gene expression and methylation data available were downloaded (Table 1) (Kresse et al., 2012; Selamat et al., 2012; Fertig et al., 2013; Kim et al., 2013; Terunuma et al., 2014). Although gene expression arrays from GEO submissions are already normalized, we created box plots to confirm it. Methylation arrays were either *Illumina 27K* (Bork et al., 2010) or *Illumina 450K* (Sandoval et al., 2011). We used *R* for our computations (R Core Team, 2012). Beta values were converted to *M*-values before normalization. Datasets were quantile normalized with the function *normalize.quantiles* implemented in the package *preprocessCore*^1^. In order to obtain a comparable list of correlations, we averaged the CpG intensity methylation for each gene within each experiment and separately for cancer and normal tissue samples. We compared the methylation-expression of those genes represented in all experiments. In the case of gene expression datasets, probes matching to the same genes were averaged so that only one vector of expression intensities was kept per gene. Only those genes that had expression and methylation measures in all the datasets were kept. Differential expression and methylation analysis were performed with *Significance Analysis for Microarrays* (SAM) (Tusher et al., 2001). SAM was run with 10,000 permutations and significance was selected by tuning the Δ value to approximately a 5% False Discovery Rate (FDR). Methylation Coefficients of Variation (a.k.a. CVs) were calculated with a modified version of the classic formula that uses interquartile ranges (a.k.a. IQR) and the median instead of the standard deviation and the media.

**Table 1:** **Origins of the data**.

**Sample type**	**Source**	**Number of controls**	**Number of cases**	**Total number**
Bladder cancer	GSE37817 (Kim et al., 2013)	6	18	24
Breast cancer	GSE39004 (Terunuma et al., 2014)	8	57	65
Head and Neck Squamous Cell Carcinoma	GSE33232 (Fertig et al., 2013)	25	44	69
Lung Adenocarcinoma	GSE32867 (Selamat et al., 2012)	57	57	114
Osteosarcoma	GSE36004 (Kresse et al., 2012)	6	19	24
Total		102	195	297

*Each line includes the type of sample, its GEO identification code, the number of cases and controls, and the total number of samples*.

### Gene expression-methylation correlation analyses

We calculated Pearson's and Spearman's correlations between methylation and gene expression intensity separately in each tumor-normal dataset and in both cases and controls. The *Wilcoxon Signed Rank Test* was used to search significant differences in correlation values between tumor and normal samples at the genome-wide level. Signed Pearson's correlation value is known not to directly bear on the size of the correlation coefficients (Goodwin and Leech, 2006), but in order to analyze differences in absolute correlations we managed potential indirect biases introduced by differences in the sample-size by randomly shuffling the datasets in the biggest group (cancer group) to the length of the smaller group (normal group). In each case we calculated the correlations genome-wide and performed the Wilcoxon Signed Rank Test 1000 times. *P*-values for differential absolute correlation genome-wide were calculated as the mean of the 1000 *P*-values obtained by random shuffling, and the mean estimates were calculated similarly. Otherwise, when no sample difference existed (e.g., in the Lung Adenocarcinoma cohort) we calculated differences in absolute correlations as though they were signed correlations. Although about 300 genes from the X chromosome were included, we observed that excluding them from the analysis did not change the results at all. In order to explore the biological plausibility of the analysis, we also tested the significance in correlation differences at the gene level between tumor and normal samples in three datasets, namely lung adenocarcinoma, head and HNSCC and breast cancer. For this, we applied Fisher's transformation to Spearman's Rank correlations. *P*-values were adjusted for the FDR.

### Determination of differentially correlated genes

DCGs are those genes whose expression and DNA methylation patterns significantly differ in cancer when compared with normal tissues. We calculated DCGs in two datasets (namely lung adenocarcinoma and HNSCC) based on the expression-average methylation correlation values for each gene. Fisher's transformation was applied to Pearson's and Spearman's correlation values and the *P*-values were calculated accordingly (Mudholkar, 2006; Myers and Sirois, 2006). Since the breast cancer methylation was analyzed with the *Illumina 450K* platform, this group included a greater number of inspected CpGs per gene. In this case, we calculated the combined Stouffer's *P*-value for all CpGs inside each gene in order to improve our use of methylation information (see Section Chromatin and Transcription Factor Binding Enrichment). This method discovered a much larger number of DCGs.

Gene Ontology (Ashburner et al., 2000), Pathways Commons (Cerami et al., 2011), and Cytoband enrichment analysis were performed with the *WEB GESTALT* tool (Wang et al., 2013). Transcription Factor Binding Sites (TFBS) and *InterPro* Protein Domain (Jones et al., 2014) enrichment analysis were performed in the *GenCodis* web browser (Tabas-Madrid et al., 2012). We used our own custom background, representing all the genes that were studied. Pathways analysis of the *KEGG* (Kanehisa, 2013) database was performed for significant miRNAs with the *DIANA-miRPath* tool (Vlachos et al., 2012). We used the “*gene union*” option, which calculates the union of targeted genes by the chosen miRNAs. This list was then used as input for pathways analysis.

### Chromatin and transcription factor binding enrichment

We wished to determine whether DCGs overlapped with any known chromatin feature. With this purpose we first annotated the genomic coordinates of each gene using the *biomaR*t package (Durinck et al., 2009). Then, we collected epigenetic data from *H1* human Embryonic Stem Cells (H1hESC), B-lymphocytes (Gm12787), hepatocellular carcinoma (HepG2), and human mammary epithelial cells (HMEC) cell lines and uploaded it to *The Genomic Hyperbrowser* (Sandve et al., 2010). These tracks were composed of the Chip-seq peak calls released following (1) the Encode Uniform Processing Pipeline, (2) the Chromatin State Segmentation based on Hidden Markov Models (HMM), (3) the Uniform DNAse 1 Hypersensitivity tracks, and (4) the histone modification tracks released by the Encode Analysis Working Group (AWG) and the Broad/MGH Encode Group, respectively. Those genomic segments whose expression-methylation changed significantly (at maximum FDR of 5%) between tumor and normal samples were labeled as case, and those whose correlation did not change significantly were chosen as controls. We applied the “*Preferential Overlap*” function of *The Genomic Hyperbrowser*, with (1) a minimal number of Monte Carlo samples of 1000, (2) a global FDR value of 0.001, (3) an alternative hypothesis of an overlap greater than expected, and (4) a null model that preserves segments of both tracks and permutes case and control assignment sequences on chip-seq tracks. Duplicate and triplicate tracks (e.g., tracks measuring the genomic binding occupancy of the same transcription factor) were handled by combining their *P*-values using the Stouffer's Z-score method. This test is a meta-analytic method closely related to the Fisher's combined probability test used to combine the result of several independent tests bearing the sample null hypothesis. Stouffer's test is sensitive to consistent departures from the null hypothesis, while Fisher's method is more sensitive to occasional departures from the null hypothesis (Abelson, 1995). Finally, *P*-value adjustment for multiple testing was performed on four separate batches of tracks according to their origin: *Encode's Uniform Pipeline TF Chip tracks*, *Encode's Chromatin State Segmentation tracks*, *AWG DNAse 1 Hypersensibility tracks* and *Broad/MGH Encode Group histone modification tracks*. *P*-values were corrected with the FDR method.

### Generalized additive models regression

In order to determine the relationship between expression-methylation Spearman's correlations and the distance to the closest TSS, we studied such relationships in the breast cancer cohort. Methylation in this study was performed with *Illumina 450k* arrays, which are suitable for genome-wide analysis. Briefly, we fitted generalized additive models (GAM) (Wood, 2011) with correlation as the dependent variable. GAM models allow for non-parametric or semi-parametric fits between the variables of interest, thus leading to better local fits. This is especially important when variables are known to have non-linear and changing relationships between them. We applied Fisher's transformation to the correlations, so that they follow approximate normal distributions. Smooth terms were calculated with the smoothing “*s*” option of the “*gam*” function in the *mgcv* R package (Wood, 2011). The thin plate regression spline “*tp*” was used, which is a low rank isotropic smoother of any number of covariates. Smoothing parameter estimation of the independent variable (absolute TSS distance) was calculated with the Generalized Cross Validation (GCV) method. Penalized regression models gain computational efficiency by choosing a relatively small basis, known as *k*. By default, we set this value to 20. Although variations in the basis have a small impact on the model, we ensured that *k*-values were not so small to cause over-smoothing by using the “*gam.check*” function. *P*-values were computed by randomly re-shuffling 20,000 times in order to calculate the null distribution of the differencing variance estimator. Low *P*-values may indicate that the basis dimension is too low. We confirmed that all models had approximately normal residuals and that the values of the estimates divided by the residual variance (a.k.a *k-index*) were close to 1. Plots were created with the *ggtools* function implemented in the *ggplot2* R package (Wickham, 2009).

### Literature search

We used *PubMed* (McEntyre and Lipman, 2001) to search for most of the bibliography cited in this paper.

## Results

### Gene expression correlation with average methylation intensity

**Figure 3:**
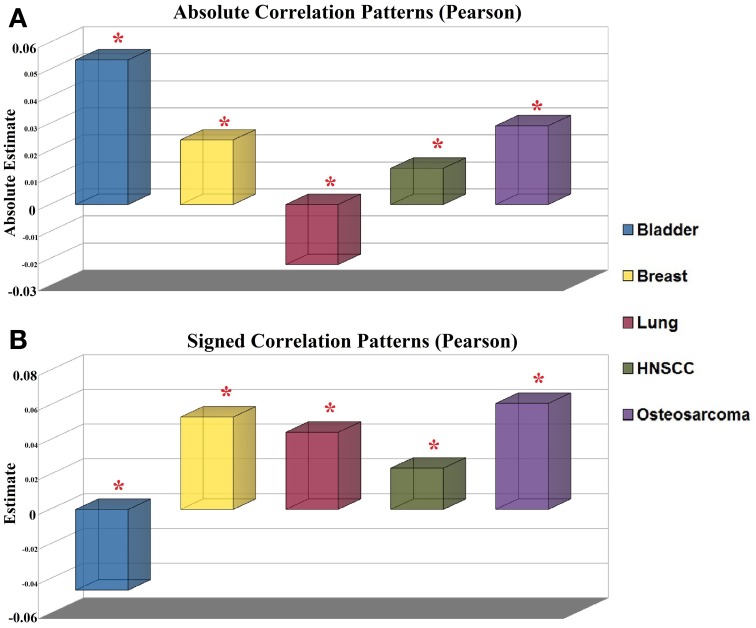
**Pearson's correlation between DNA methylation and gene expression in five different types of cancer-normal tissues. (A)** Reported *estimates* are the mean of the 95% CI obtained after calculating signed Spearman's correlations in 1000 random subsets of the same population size. Asterisks indicate significant differences at a 10% FDR. **(B)** Same as above for signed Pearson's correlation values.

We evaluated the relationship between DNA methylation and expression across more than 6200 genes in all the tumor and normal tissue samples (Supplementary Table 1). We observed significant differences of absolute Pearson's correlation in all the five study groups (10% FDR; Table 2 and Figure 3). Spearman's correlation reduced the level of significant datasets to 4 of 5 (10% FDR), since in this case the breast cancer dataset was not significant (*Q*-value = 0.214; Table 2). Notably, most of these absolute values were higher in normal tissues than in cancer, except for the Lung Adenocarcinoma dataset. Similarly, the signed correlation values revealed significant Pearson's and Spearman's differences between tumor and normal samples in all study cohorts (FDR <0.1%; Table 2 and Figure 3). Four of these groups had a more positive correlation in normal samples, and in the Bladder Cancer cohort the contrary holds. These results suggest that common correlation differences exist in cancer tissues.

**Table 2:** **Correlation results in the 5 tumor-normal datasets**.

	**Bladder**	**Breast**	**Lung**	**HNSCC**	**Osteosarcoma**
**(A) PEARSON'S CORRELATION RESULTS**					
Absolute estimate	0.053	0.023	−0.022	0.013	0.029
Absolute mean *P*-value	1.98 × 10^−12^	0.061	1.72 × 10^−35^	0.033	6.2 × 10^−4^
FDR *Q*-value	4.95 × 10^−12^	0.061	8.65 × 10^−35^	0.041	1.00 × 10^−3^
	1000 random permutations on datasets of equal size				
	Positive values are higher in normal samples				
Estimate	−0.046	0.053	0.044	0.024	0.06
*P*-value	6.35 × 10^−10^	2.20 × 10^−16^	2.20 × 10^−16^	1.09 × 10^−9^	7.59 × 10^−15^
FDR *Q*-value	7.93 × 10^−10^	5.50 × 10^−16^	5.50 × 10^−16^	1.09 × 10^−9^	1.26 × 10^−14^
**(B) SPEARMAN'S CORRELATION RESULTS**					
Absolute Estimate	0.023	−0.006	−0.018	0.009	0.033
Absolute Mean *P*-value	0.0082	0.21	1.43 × 10^−26^	0.061	8.04 × 10^−7^
FDR *Q*-value	0.014	0.21	7.15 × 10^−26^	0.077	2.01 × 10^−6^
	1000 random permutations on datasets of equal size				
	Positive values are higher in normal samples				
Estimate	−0.036	0.049	0.038	0.018	0.055
*P*-value	2.37 × 10^−7^	2.20 × 10^−16^	2.20 × 10^−16^	7.51 × 10^−7^	8.69 × 10^−13^
FDR *Q*-value	2.96 × 10^−7^	5.50 × 10^−16^	5.50 × 10^−16^	7.51 × 10^−7^	1.44 × 10^−12^

*(A) Person's correlations for absolute correlation (upper table) and signed correlation (lower table) differences. Reported *estimates* are the mean of the 95% CI obtained after calculating correlations in 1000 random subsets of the same population size. (B) Spearman's correlations for absolute (upper table) and signed (lower table) correlation differences. Reported *estimates* are the mean of the 95% CI*.

### Differentially correlated genes in lung adenocarcinoma and HNSCC

By applying Fisher's transformation to Spearman's correlations in the lung adenocarcinoma and HNSCC cohorts we discovered genes whose average methylation-gene expression correlation varies significantly between tumor and normal samples (a.k.a. DCGs). In the case of lung adenocarcinoma, we found 43 DCGs at a 10% FDR (Supplementary Table 1), and 16 at a 5% FDR. The most significant finding was that of *CD40* (*Q*-value = 3.36 × 10^−4^), whose Spearman's correlation was much more negative in adenocarcinoma samples (rho = −0.66) than in matched normal controls (rho = 0.25), and whose expression was significantly higher in normal samples (*P*-value = 2.82 × 10^−4^). Remarkably, the other top findings were *CEACAM5* (*Q*-value = 0.016), *MYEOV*, *SLMAP*, and *TM4SF18* (*Q*-value = 0.017), *CD300LG* (*Q*-value = 0.02), and *ENOSF1*, *MFAP5*, *PKIA*, *SFRP1*, and *ZFP3* (*Q*-value = 0.026). In the case of HNSCC we found 15 genes significant at a 10% FDR (Supplementary Table 1), five of which had *Q*-values below 0.05. These were *IL12RB2*, *PTHLH*, *SLC7A11* (*Q*-value = 0.013), *SERPINB7* (*Q*-value = 0.029), and *CRAT* (*Q*-value = 0.046). Of particular interest is the fact that *FGF11* was present in the two DCGs lists (lung adenocarcinoma *Q*-value = 0.037, HNCC *Q*-value = 0.053), which is an important finding when considering that the methylation array platform in these two cases was the same (*Illumina 27K*). Moreover, this gene remained nearly significantly differentially correlated in breast cancer at a 5% FDR (*Q*-value = 0.067; *vide infra*). Overall, the correlation tendency of DCGs was more negative in cancer than in normal samples. About 67% and 80% of these genes had significantly lower correlations in lung adenocarcinoma and HNSCC; respectively.

We combined all the significant genes in the two lists (10% FDR) and performed gene ontology analysis versus its own background. This revealed significant enrichments of the selected 58 genes in the following biological processes.: “*cellular response to radiation*” (*Q*-value = 2.01 × 10^−2^), “*convergent extension involved in axis elongation*” (*Q*-value = 2.92 × 10^−2^), “*negative regulation of non-canonical Wnt receptor signaling pathway*” (*Q*-value = 2.92 × 10^−2^), “*negative regulation of planar cell polarity pathway involved in axis elongation*” (*Q*-value = 2.01 × 10^−2^), “*negative regulation of endopeptidase activity*” (*Q*-value = 2.01 × 10^−2^), “*negative regulation of B cell apoptotic process*” (*Q*-value = 4.68 × 10^−2^), and “*negative regulation of peptidyl-tyrosine phosphorylation*” (*Q*-value = 2.68 × 10^−2^) (Supplementary Figure 1). Significant enrichments in two protein interaction modules were also observed. The first one contains two DCGs (namely *ZC3H11A* and *CRAT*; *Q*-value = 0.019) and is related to “*fatty acid beta-oxidation using acyl-CoA oxidase*,” “*peroxisomal matrix*” and “*C-acetyltransferase activity*” processes. The second one contains five DCGs (namely *CD40*, *NOX4*, *EDARADD*, *BCL10*, *IL1R2*; *Q*-value = 0.039) and is related to “*regulation of interferon-beta production*,” “*CD40 receptor complex*,” and “*tumor necrosis factor receptor superfamily*” processes.

### Focused analysis on breast cancer

In order to further determine the potential impact that DCGs have in cancer, we decided to investigate the distribution of expression-methylation Spearman's correlations in the breast cancer dataset, since this dataset is based on a high-density array of CpG methylation probes. For this, we used Fisher's transformed Spearman's correlation values, which are distributed normally and allow calculating *P*-values for differences in correlations. The Stouffer test was used to combine all *P*-values into a single, gene-level *P*-value. The presence of significant autocorrelation between CpG correlations can increase the Type I error of the Stouffer test. Using the Mantel test, we observed no significant autocorrelation within 200 and 1000 base pairs from the closest TSS (10% FDR). Similarly, no significant autocorrelation was observed between CpGs falling further than 1000 base pairs upstream and downstream from the closest TSS (10% FDR). Thus, we can reasonably assume that the Type I error is not artificially inflated in this study.

#### DNA methylation correlation with gene expression as a function of transcription start site distance

**Figure 4:**
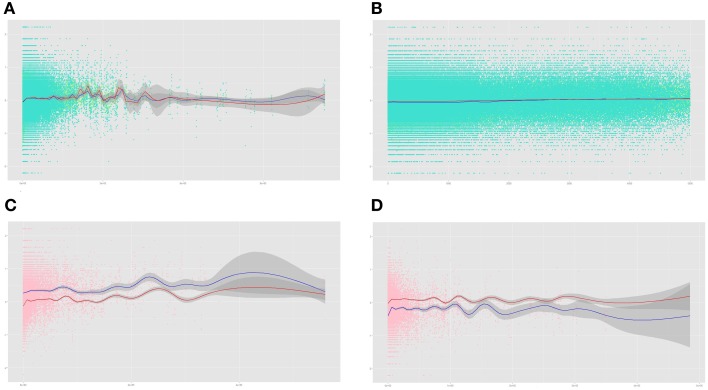
**GAM regression of breast correlations as a function of absolute distance to the closest TSS. (A)** GAM regression of Fisher's Z-Transformed Spearman's Correlations vs. absolute TSS distance. The blue and the red lines are the regressions of correlations in cancer and normal breast, respectively. Dark gray areas indicate 95% CI. Turquoise and light green dots are Z-correlations in cancer and normal breast, respectively. **(B)** Same as the previous section but focused in the 5000 bp region from the closest TSS. **(C)** GAM regression of DCGs with lower correlations in cancer than in normal breast. The blue and red regression lines correspond to normal and cancer breast, respectively. Pink dots indicate Spearman's correlation Z-scores in normal breast. (**D**) Same as in the previous section for those DCGs with higher correlations in cancer than in normal breast.

We created a GAM model to inspect the relationships between CpG methylation intensity and proximal gene expression as a function of the distance to the closest TSS. The model showed that CpGs in the immediate vicinity of the closest TSS showed negative correlations with gene expression in both cancer and normal samples, but this pattern was the inverse in more distant points (Figure 4A; Supplementary Figures 2, 3). For example, we found that approximately from 5000 bp to the closest TSS until 400,000 bp the model was consistent with more positive than negative correlations in both cancer and normal tissues, reaching points of high statistical significance. Very curiously, a zoom into the first 5000 bp from the TSS revealed that the two models are similar but the cancer model was slightly more negative during the first 2000 bp (Figure 4B; Supplementary Figures 4, 5). Although the difference was small, the 95% confidence intervals of both normal and cancer samples do not overlap in this region, and the *T*-test confirmed a significantly more positive correlation in normal samples (*P*-value = 5.99 × 10^−69^, 95% CI [0.017, 0.021]), whilst outside this 5000 bp region the correlations did not show such a strong effect (*P*-value = 0.021, 95% CI [7.00 × 10^−4^, 9.00 × 10^−4^]). We defined the correlation ratio as the ratio of correlation Z scores (*Normal Z score/Cancer Z score*). We observed that within the first 5000 base pairs the ratio is significantly more negative than outside this region (Wilcoxon Rank Sum Test *P*-value = 2.06 × 10^−18^, 95% CI [−0.204, −0.129], mean value within 5000 bp = 0.166, mean outside 5000 bp = 0.39), indicating that correlation values are more divergent and that more correlation sign shifts exist within the 5000 bp than outside (42.00% vs. 39.88%).

#### Determination of expression-methylation differentially correlated genes in breast cancer

In order to characterize those genes with changing expression-methylation correlations, we combined one-tailed Fisher's *P*-values at the gene level and identified groups of genes whose correlation patterns varied between cancer and normal cases significantly. We observed 1662 DCGs at a 5% FDR (Supplementary Table 2). Notably, no correlation was observed between the *P*-values and the number of CpGs covering each gene (Spearman's correlation = 0.004 and −0.004, *P*-value = 0.633 for the lists of genes more positively and more negatively correlated in cancer vs. normal, respectively). However, a significant enrichment of DCGs vs. background genes in GC content was observed (*P*-value = 2.91 × 10^−19^, Wilcox), which is an inherent bias due to the Stouffer test and the characteristics of the methylation arrays. The top DCGs can be consulted in Tables 3, 4.

**Table 3:** **Top 20 genes in the list of DCGs with higher correlation in breast cancer**.

**Gene name**	**Stouffer's *P*-value**	**Number of CpGs**	**FDR**	**Bonferroni**
*DOK7*	0	72	0	0
*CACNA1H*	0	185	0	0
*IRX4*	0	183	0	0
*ZIC4*	0	91	0	0
*PXDNL*	0	15	0	0
*CLCN7*	0	64	0	0
*LMF1*	0	256	0	0
*GPT*	0	28	0	0
*NARS*	0	14	0	0
*MUC5B*	0	72	0	0
*ARHGEF7*	0	46	0	0
*FLRT2*	0	18	0	0
*KRT85*	0	12	0	0
*OSBPL8*	0	14	0	0
*NID2*	1.44 × 10^−15^	19	1.37 × 10^−12^	2.05 × 10^−11^
*TRAP1*	3.76 × 10^−14^	50	3.34 × 10^−11^	5.35 × 10^−10^
*UBE2MP1*	6.51 × 10^−14^	62	5.44 × 10^−11^	9.24 × 10^−10^
*KNDC1*	6.99 × 10^−14^	128	5.52 × 10^−11^	9.93 × 10^−10^
*GNMT*	9.66 × 10^−14^	37	7.22 × 10^−11^	1.37 × 10^−9^

**Table 4:** **Top 20 genes in the list of DCGs with higher correlation in normal cancer**.

**Gene names**	**Stouffer's *P*-value**	**Number of CpGs**	**FDR**	**Bonferroni**
*NR2F2*	0	163	0	0
*SMOC2*	0	179	0	0
*SIM1*	0	94	0	0
*TNFRSF25*	0	30	0	0
*CXXC1*	0	21	0	0
*RASA3*	0	329	0	0
*MIR153-2*	0	316	0	0
*TBX5*	0	53	0	0
*PCDH8*	0	36	0	0
*TMEM132D*	0	38	0	0
*PADI2*	0	13	0	0
*RASSF1*	0	55	0	0
*PCDHB4*	0	21	0	0
*ALPI*	0	22	0	0
*SCAF1*	0	12	0	0
*ERMAP*	0	12	0	0
*NUDT10*	3.77 × 10^−15^	42	3.15 × 10^−12^	5.36 × 10^−11^
*CRABP2*	4.55 × 10^−15^	17	3.59 × 10^−12^	6.47 × 10^−11^
*ADAMTS2*	5.11 × 10^−15^	52	3.63 × 10^−12^	7.25 × 10^−11^

#### DCGs correlation distribution

We considered it interesting to analyze the patterns of expression-methylation correlation of the DCGs as a function of their distance to the closest TSS in normal and neoplastic breast tissues. GAM regression of the DCGs more positively correlated in cancer provided evidence for a general difference, which is more abrupt in the TSS region (Figure 4D, Supplementary Figures 6, 7). A similar but opposite trend can be observed in the model of the genes more negatively correlated in cancer (Figure 4C, Supplementary Figures 8, 9). Thus, our pipeline efficiently identifies those genes whose correlation patterns vary constantly at the gene level.

#### DCGs methylation and expression patterns

In order to inspect possible differences in methylation and expression between cancer and normal samples, we calculated the mean and standard deviation for each CpG, with a special focus on CpGs corresponding to DCGs. The mean methylation intensity was significantly lower in normal samples (*P*-value < 2.2 × 10^−16^, 95% CI [−0.045, −0.0436]; Wilcoxon signed rank test (a.k.a ***Wilcox***)) and in DCGs (*P*-value < 2.2 × 10^−16^, 95% CI [−0.063, −0.058]; Wilcox). Interestingly, the average methylation intensity within DCGs was substantially higher compared to the rest of the genome in both cancer (*P*-value < 2.2 × 10^−16^, 95% CI [0.55, 0.60]; Wilcox) and normal tissues (*P*-value < 2.2 × 10^−16^, 95% CI [0.51, 0.56]; Wilcox). Standard deviation was substantially higher inside DCGs compared to the rest of the analyzed genome both in normal (*P*-value < 2.2 × 10^−16^, 95% CI [0.047, 0.050]; Wilcox) and in cancer tissues (*P*-value < 2.2 × 10^−16^, 95% CI [0.098, 0.104]; Wilcox). In order to compare possible differences in variability of DCGs and background genes we calculated the CVs on methylation β-values. The data indicate significantly higher variability in cancer both at DCGs and background genes (*P*-values <2.2 × 10^−16^), and although the difference is higher among DCGs, the median difference in CVs is only 2.1% higher (95% CI for DCGs [9.43, 9.67]; 95% CI for background genes [7.36, 7.45]; Wilcox). Thus, there is no clear reason to believe that increased variability affects the determination of DCGs.

To determine possible promoter-dependent differences in methylation, we split the data into promoter-associated CpGs (at less than 1 Kb from the TSS) and in non-promoter associated CpGs. Average methylation of CpGs was found to be significantly higher in promoter-associated CpGs of DCGs with respect to their counterparts in the rest of the genome in both normal and cancer data (Wilcox *P*-values < 2.2 × 10^−16^, 95% CI [0.404, 0.453] and [0.462, 0.515], respectively), whilst average methylation was lower in non-promoter CpGs compared with their genomic homologs in both cases (Wilcox *P*-values < 2.2 × 10^−16^; 95% CI [−0.158, −0.099] and [−0.154, −0.097], respectively). Interestingly, promoter associated and non-promoter associated CpGs were significantly hypomethylated in normal compared to cancer breast (*P*-value < 2.2 × 10^−16^; Wilcox). Using *SAM* we identified a general trend of DCGs promoter hypermethylation in cancer and 138 genes whose average promoter methylation was significantly higher in cancer at a 5% FDR (Supplementary Figure 10A). Similarly, average methylation at non promoter-associated regions showed a trend toward hypermethylation in 112 DCGs (5% FDR), but no significant differences were associated with the remaining 93% of the DCGs (Supplementary Figure 10B). Nevertheless, using the Wilcoxon Signed Rank Test we could not identify a single gene whose average methylation at its promoter or outside it was statistically significant at a 10% FDR. Similarly, we found no CpGs that did so individually, although 61% of the location parameter estimates were preferentially biased toward a higher methylation in the cancer group. Finally, promoter associated and non-promoter associated CpGs were more variable in DCGs than in the rest of the genome both in normal and in cancer (*P*-values < 2.2 × 10^−16^; Wilcox).

Average gene expression was significantly higher in cancer genome-wide and in DCGs (Wilcox *P*-values = 1.75 × 10^−53^ and 3.445 × 10^−5^, respectively). However, among all the DCGs no significant difference in expression could be observed at a 5% FDR using *SAM;* and only 50 genes ranked significant at a 10% FDR, with a clear trend of these to be more expressed in normal breast (Supplementary Figure 10C). The average expression of DCGs was significantly higher compared to the rest of the analyzed genome in normal and cancer tissues (Wilcox *P*-value = 2.39 × 10^−7^ and 1.21 × 10^−6^, respectively; 95% CI [0.131, 0.291] and [0.116, 0.276], respectively). Nevertheless, the expression of DCGs is not significantly more variable than the rest of the genome neither among normal samples (Wilcox *P*-value = 0.173) nor among cancer tissues (Wilcox *P*-value = 0.106).

#### Separate analysis of DCGs methylation and expression patterns by correlation difference sign

Since the 1662 DCGs contain genes that vary in different directions we analyzed those genes whose correlation was more negative in cancer (1001 in total) separately from the rest (661 genes). By comparing the average methylation for each CpG, we observed that both groups were hypermethylated with respect to the rest of the genome in normal and cancer tissues (*P*-values < 2.2 × 10^−16^; Wilcox). Although CpG average methylation was greater in cancer in both groups of DCGs (*P*-values < 2.2 × 10^−16^; Wilcox), this measure was more significant in those genes with more positive correlations in cancer than in those with more negative correlations in cancer (Wilcox *P*-values = 3.85 × 10^−10^ and 2.30 × 10^−7^ in cancer and normal samples, respectively). Average expression was greater among genes with significantly higher correlation values in cancer than in the rest of the genome (Wilcox *P*-values = 0.0025 and 0.0027 for normal and cancer, respectively), but no difference was observed for those genes with lower correlation in cancer (Wilcox *P*-values = 0.204 and 0.263 for normal and cancer groups, respectively). However, no significant difference in expression was detected for both groups of DCGs between cancer and normal breast tissue (Wilcox *P*-values = 0.857 and 0.912 for genes with higher and lower correlations in cancer, respectively).

#### Gene ontology and pathways analysis of DCGs

We searched for the enrichment of the DCGs in gene ontology terms (Supplementary Figure 11). DCGs are notably enriched in various biological processes, with a special emphasis in “*cell differentiation*” (*Q*-value = 5.09 × 10^−11^), “*system development*” (*Q*-value = 9.24 × 10^−11^), “*regulation of cell proliferation*” (*Q*-value = 1.92 × 10^−5^), “*organ morphogenesis*” (*Q*-value = 3.36 × 10^−6^), “*neuron development*” (*Q*-value = 6.62 × 10^−6^), and “*embryonic skeletal system morphogenesis*” (*Q*-value = 3.12 × 10^−5^). Similarly, DCGs were enriched in “*phospholipid binding*” (*Q*-value = 3.35 × 10^−2^), “*HMG box domain binding*” (*Q*-value = 4.79 × 10^−2^), “*neurotrophin receptor binding*” (*Q*-value = 1.89 × 10^−2^), “*regulatory region DNA binding*” (*Q*-value = 1.90 × 10^−3^), “*sequence-specific DNA binding*” (*Q*-value = 2.55 × 10^−10^), “*nucleic acid binding transcription factor activity*” (*Q*-value = 1.18 × 10^−5^), and in “*transmembrane signaling receptor activity*” (*Q*-value = 6.80 × 10^−3^). DCGs are also enriched in proteins located at the extracellular region (*Q*-value = 8.00 × 10^−3^) and in the plasma membrane (*Q*-value = 6.5 × 10^−3^). Fifty-five DCGs contain *Homeobox domains*, which implies a highly significant enrichment (*Q*-value = 7.85 × 10^−8^); and 44 genes contain *Pleckstrin homology domains* (*Q*-value = 0.048, hyper; Supplementary Table 3). Chromosome Xq was the only genomic region enriched in DCGs at a 5% FDR (*Q*-value = 2.9 × 10^−3^) (Supplementary Table 4). Another two cytobands were significantly enriched at a 10% FDR (namely 5q34 and 18q23). We also searched for pathway enrichments in the *Pathways Commons* database, which retrieved a list of 51 significantly enriched pathways (5% FDR, hypergeometric, Supplementary Table 5). These were “*PAR1-mediated thrombin signaling events*,” “*Integrin family cell surface interactions*,” “*Class I PI3K signaling events mediated by Akt*,” “*ErbB1 downstream signaling*,” “*Arf6 signaling events*,” “*EGFR-dependent Endothelin signaling events*,” “*Internalization of ErbB1*,” “*Signal Transduction*,” “*mTOR signaling pathway*,” “*VEGF and VEGFR signaling network*,” “*Nectin adhesion pathway*,” “*CDC42 signaling events*,” “*Glypican pathway*,” “*Proteoglycan syndecan-mediated signaling events*,” “*Thrombin/protease-activated receptor (PAR) pathway*,” “*EGF receptor (ErbB1) signaling pathway*,” “*Regulation of CDC42 activity*,” “*IFN-γ pathway*,” “*Arf6 trafficking events*,” “*β1 integrin cell surface interactions*,” “*Urokinase-type plasminogen activator (uPA) and uPAR-mediated signaling*,” “*ErbB receptor signaling network*,” “*IL5-mediated signaling events*,” “*PDGFR-β signaling pathway*,” “*Signaling events mediated by focal adhesion kinase*,” “*S1P1 pathway*,” “*Hemostasis*,” “*Signaling events mediated by VEGFR1 and VEGFR2*,” “*Insulin Pathway*,” “*Thromboxane A2 receptor signaling*,” “*PDGF receptor signaling network*,” “*IL3-mediated signaling events*,” “*GMCSF-mediated signaling events*,” “*Platelet activation, signaling and aggregation*,” “*Validated transcriptional targets of AP1 family members Fra1 and Fra2*,” “*Glypican 1 network*,” “*Class I PI3K signaling events*,” “*Trk receptor signaling mediated by PI3K and PLC- γ*,” “*Arf6 downstream pathway*,” “*LKB1 signaling events*,” “*Sphingosine 1-phosphate (S1P) pathway*,” “*IGF1 pathway*,” “*Syndecan-1-mediated signaling events*,” “*Signaling events mediated by Hepatocyte Growth Factor Receptor (c-Met)*,” “*Neurotrophic factor-mediated Trk receptor signaling*,” “*Endothelins*,” “*α9β1 integrin signaling events*,” “*Signaling by GPCR*,” “*TRAIL signaling pathway*,” “*Signaling events mediated by PTP1B*,” and “*Plasma membrane estrogen receptor signaling*.”

Enrichment in TFBS was significant for 62 TFs (Supplementary Table 6). The two top findings were TCF3 and LEF1 (*Q*-values = 1.38 × 10^−4^ and 5.53 × 10^−4^, respectively). Similarly, we found a significant enrichment in miR-574-5p, miR-637, and miR-663 target genes (*Q*-values < 0.05). We searched for enrichment of all significant miRNAs-target genes in KEGG pathways (Supplementary Table 7). The resulting data indicates that these miRNAs regulate the following pathways: “*Biosynthesis of unsaturated fatty acids*” (*Q*-value = 2.12 × 10^−13^), “*TGF-β signaling pathway*” (*Q*-value = 8.55 × 10^−12^), “*p53 signaling pathway*” (*Q*-value = 4.76 × 10^−8^), “*Pathways in cancer*” (*Q*-value = 6.41 × 10^−8^), “*Renin-angiotensin system*” (*Q*-value = 2.22 × 10^−5^), “*MAPK signaling pathway*” (*Q*-value = 3.99 × 10^−5^), “*GABAergic synapse*” (*Q*-value = 1.43 × 10^−4^), “*Sphingolipid metabolism*” (*Q*-value = 2.84 × 10^−4^), “*Prostate cancer*” (*Q*-value = 4.81 × 10^−4^), “*Renal cell carcinoma*” (*Q*-value = 7.38 × 10^−4^), “*PI3K-Akt signaling pathway*” (*Q*-value = 1.04 × 10^−3^), “*Focal adhesion*” (*Q*-value = 1.60 × 10^−3^), “*Adipocytokine signaling pathway*” (*Q*-value = 2.09 × 10^−3^), “*Transcriptional misregulation in cancer*” (*Q*-value = 4.02 × 10^−3^), “*Maturity onset diabetes of the young*” (*Q*-value = 8.54 × 10^−3^), “*Small cell lung cancer*” (*Q*-value = 8.54 × 10^−3^), “*Ubiquitin mediated proteolysis*” (*Q*-value = 1.57 × 10^−3^), “*Circadian rhythm*” (*Q*-value = 1.57 × 10^−2^), “*mTOR signaling pathway*” (*Q*-value = 2.20 × 10^−2^), “*Hedgehog signaling pathway*” (*Q*-value = 2.20 × 10^−2^), “*Neurotrophin signaling pathway*” (*Q*-value = 2.64 × 10^−2^), “*Acute Myeloid Leukemia*” (*Q*-value = 3.68 × 10^−2^), and “*Lysine degradation*” (*Q*-value = 4.23 × 10^−2^).

#### DCGs enrichment in transcription factor (TF) peaks, chromatin modifications, and chromatin state in model cell lines

By comparing uniformly processed genomic tracks for a preferential overlap of DCGs in various TF peaks we found marked enrichments (*P*-value < 9.9 × 10^−4^) in EZH2 in four cell lines, namely Gm12878, H1hESC, HEPG2, and HMEC. Notoriously, the cell line that presented more significant overlaps was H1hESC, in which 11 TF tracks ranked significant (*Q*-value < 0.05). These were BCL11A, CTBP2, CTCF, EGR1, EZH2, GABPA, NANOG, RAD21, SUZ12, TCF2, and ZNF143. Only one track was available for SUZ12, but the enrichment was highly significant (*P*-value < 9.9 × 10^−4^), which along with the EZH2 tracks makes plausible a preferential binding of PRC2 in these genomic regions. Twenty-seven tracks were available for H1hESC, HEPG2, and Gm12787 cell lines. Stouffer's corrected values revealed significant overlaps of DCGs with CTCF and EZH2 tracks (*Q*-values = 1.69 × 10^−4^ and 1.17 × 10^−6^, respectively). H1hESC were also enriched in H2A.Z, H3K27me3, H3K4me1, and H3K4me2 tracks. Stouffer merged *P*-values across Gm12878, HMEC, HEPG2, and H1hESC revealed significant enrichments in H3K27me3 and H3K9me3 chromatin marks (*Q*-values = 6.81 × 10^−9^ and 0.027, respectively). A similar procedure was followed with Chromatin State Segmentation tracks, which revealed significant enrichments in “*Repressed*” (*Q*-value = 1.80 × 10^−8^), “*Heterochromatin*” (*Q*-value = 1.80 × 10^−8^), “*Poised Promoter*” (*Q*-value = 4.77 × 10^−9^), and “*Insulator*” (*Q*-value = 0.024) chromatin domains. Finally, DNA Hypersensibility regions preferentially overlapped with DCGs in H1hESC and HMEC cell lines (*P*-values = 9.9 × 10^−4^ and 0.002, respectively), but not in Gm12878 and HEPG2. Results of these experiments can be consulted in Supplementary Table 8.

#### Enrichment of DCGs in partially methylated domains, epigenetic islands, lamina-associated domains, imprinted genes, HOTAIR target genes and somatic copy number aberrations

Since hypermethylation of promoters and hypomethylation of non-promoter associated regions is a common feature of Partially Methylated Domains (PMDs Hon et al., 2012), we tested whether the overlap between DCGs with previously defined PMDs and heavily methylated domains (HMDs) (Schroeder et al., 2011) was higher than the overlap of background genes. Note that these PMDs and HMDs were common to placenta, neurons and fibroblasts. We found strong evidence supporting an enrichment in PMDs (*P*-value = 2.04 × 10^−6^, hypergeometric) but no significant overlap was observed with HMDs (*P*-value = 1, hypergeometric). The average expression of those DCGs overlapping with known PMDs was nearly significantly lower than the rest of the DCGs in normal breast (*P*-value = 0.0527, Wilcox), but not in breast cancer (*P*-value = 0.1904, Wilcox). DCGs overlapping known PMDs are hypermethylated compared with non-overlapping DCGs in normal and cancerous breast (*P*-values < 2.2 × 10^−16^; Wilcox). Indeed, PMD-overlapping DCGs tend to be hypermethylated in cancer compared with normal breast (*P*-value = 5.46 × 10^−150^; Wilcox). Although non-PMD DCGs are also hypermethylated in cancer, PMD-overlapping DCGs are more strongly methylated than non-PMD DCGs (PMD DCGs 95% CI [−0.266, −0.233]; non-PMD DCGs 95% CI [−0.044, −0.042]; Wilcox).

Similarly, we obtained a list of 93 imprinted human genes from the *geneimprint database* (Jirtle, 1997), of which 53 were among the list of genes in this study. We discovered a marked enrichment of DCGs in imprinted genes (*P*-value = 1.06 × 10^−9^; hypergeometric). Feinberg et al. (Wen et al., 2012) described the existence of euchromatin islands (EIs) within heterochromatin domains, and since these were enriched in CTCF binding and DNAse I hypersensitivity regions, we decided to test for significant overlaps between our DCGs coordinates, their background regions and all the EI coordinates found in 5 different cell lines. Once again, we observed a mildly significant enrichment among DCGs (*P*-value = 0.017). DCGs are enriched in Homeobox genes and *HOTAIR* is a non-coding RNA derived from a *HOX* gene and involved in the aberrant gene targeting of PRC2 during cancer progression (Gupta et al., 2010). By comparing background genes and DCGs with a list of genes bound to PRC2 after *HOTAIR* induction in breast cancer cells (Gupta et al., 2010), we observed a significant enrichment of these genes on the DCG category (*P*-value = 6.35 × 10^−5^; hypergeometric).

Finally, DCGs are also enriched in CpG islands and GC content compared with their background genes (*P*-value = 9.99 × 10^−5^ and 2.2 × 10^−16^, Wilcox). Due to the fact that DNA G Quadruplexes (a.k.a. G4) are known to influence DNA methylation (Halder et al., 2010) and gene expression (Agarwal et al., 2014), we analyzed the overlap between DNA G4 motif sequences reported by Balasubramanian et al. (Huppert and Balasubramanian, 2007) and DCGs, observing a very significant enrichment (*P*-value < 9.99 × 10^−4^). Nevertheless, this can be a statistical artifact due to the enrichment of DCGs in GC content and must be interpreted with caution. Finally, we also compared DCGs loci with regions of focal Somatic Copy Number Aberrations (SCNAs) enriched in cancer. These SCNAs were discovered after analyzing 3131 and 1480 cancer and normal tissue samples, respectively (Beroukhim et al., 2010). DCGs were found to be mildly enriched in cancer-specific SCNA coordinates (*P*-value = 0.0128).

## Discussion

The effect of DNA methylation on gene expression is widely known to depend on its relative location with respect to the TSS of each gene (Brenet et al., 2011; Lou et al., 2014). Normally, gene body methylation correlates positively with gene expression, whilst methylation at the TSS does the opposite (Lou et al., 2014). Although the existence of different correlations has been described elsewhere, systematic genome-wide differential trends in correlations between groups have not been systematically described until now. The aim of this study is to help in moving from the idea that “*DNA methylation restricts expression*” to one more elaborate like “*DNA methylation, which is a repressive mark, has varying effects on gene expression as a result of the interaction with many other factors. It is necessary for science to study and quantify these phenomena in order to better understand human biology and health*.” In this article we have described intriguing differences between cancer and normal samples genome-wide. Although the functional consequences of these findings are still obscure, an important point to consider is that DNA methylation can drive alternative promoter use and the production of different transcript isoforms (Wang et al., 2011). It has also been suggested that intragenic methylation suppresses intragenic initiation of transcription, thereby limiting the expression of interfering RNAs transcribed within larger genes (Jjingo et al., 2012). Both mechanisms can have an effect on tumor biology, and they may help to explain the observed differences that we report. Similarly, spurious effects of SCNAs in methylation arrays have been described (Houseman et al., 2009). For example, haploid and aneuploid regions affect the methylation estimates. Thus, the presence of SCNAs in cancer samples can influence the variability observed in correlations. Indeed, we found significant enrichments of breast DCGs in Xq, 5q34, and 18q23 chromosomal regions, which are frequenctly deleted in various types of breast cancer (Huang et al., 1995; Yu et al., 2009). These are probably indicating SCNAs, and although they probably affect a minority of the detected DCGs, future studies should address these effects.

We detected the presence of DCGs in lung adenocarcinoma and HNSCC. In the case of HNSCC, we found 15 DCGs at a 10% FDR. The top genes were *IL12RB2*, *PTHLH*, and *SCL7A11*, which are all involved in cancer biology (Liu et al., 2013; Suzuki et al., 2013; Urosevic et al., 2014). *CD40* and *CEACAM5* were the top DCGs in the lung adenocarcinoma group, and both play important roles in cancer biology (Blumenthal et al., 2005; Govindan et al., 2009; Zheng et al., 2011; Rakhmilevich et al., 2012; Creelan et al., 2013; Moran et al., 2013). By focusing our research on the case of breast cancer, we discovered the existence of 1662 DCGs, which are enriched in cancer and differentiation-related pathways. The subtle hypermethylation observed in breast DCGs promoters, along with the hypomethylation status outside it, resembles PMDs. Consistently, a significant enrichment of DCGs in previously defined PMDs was observed (Schroeder et al., 2011). PMDs have been described in MCF-7 breast cancer cell lines, where large regions of hypomethylation exist (Shann et al., 2008). Ren et al. (Hon et al., 2012) demonstrated (1) that PMDs in breast cancer are associated with hypomethylated gene bodies and lower transcript abundance; and (2) that PMD-hypomethylated gene bodies overlap with repressive chromatin marks (H3K27me3 and H3K9me3) independently of the methylation status of their promoters. Since breast DCGs are enriched in cancer and cell differentiation pathways, as well as in bivalent and heterochromatic genomic domains, it is tempting to speculate that a PMD reorganization process occurs frequently at DCGs loci during carcinogenesis which mediates subtle regulation of cancer gene expression.

Nevertheless, the proportion of DCGs known to be PMDs is quite small (<10%). We also observed that DCGs are significantly enriched in EZH2 and SUZ12 binding in different cell lines, along with significant enrichments in repressive and bivalent chromatin marks. EZH2 and SUZ12 are the major components of the Polycomb Repressive Complex 2 (PRC2), which mediates transcriptional repression partially through histone methylation (Morey and Helin, 2010). Curiously, DNA methylation and PRC2-depedent histone methylation are mechanistically linked. For example, EZH2 recruits DNA methyltransferases to Polycomb-silenced genes inducing *de novo* DNA methylation patterns, a fact which is especially important in stable gene silencing of cancer cells (Morey and Helin, 2010). However, additional gene silencing mediated by Polycomb can occur in the absence of DNA methylation (Gieni and Hendzel, 2009). For example, PRC2 and H3K27me3 are known to cooperate with H3K9 methylation to maintain gene silencing by a mechanism that involves HP1α anchorage at chromatin (Boros et al., 2014). Furthermore, although EZH2 methylates H3K27 and is associated with breast cancer progression, the disposition of both factors occurs independently in mammary neoplasia (Bae et al., 2014). This is in line with the activating effect that EZH2 induces on the *NOTCH1* gene (Gonzalez et al., 2014), which triggers the expansion of cancer stem cells through a repressive-independent mechanism. Moreover, EZH2 is also involved in the regulation of *HOX* genes (Wu et al., 2008), which are very enriched within breast DCGs. A relevant effect of EZH2 on DNA methylation was observed precisely at the *HOX* loci in mantle cell lymphoma (MCL) and chronic lymphocytic leukemia (CLL) (Kanduri et al., 2013). EZH2 was observed to trigger H3K27 methylation in both tumoral samples, but a hypermethylation event that led to long-term repression occurred specifically in MCL after *EZH2* over-expression. Finally, an important gene at the *HOXC* locus involved in PRC2-mediated repression is *HOTAIR*, which encodes a large intervening noncoding RNA (lincRNA) associated with breast cancer prognosis (Sørensen et al., 2013). *HOTAIR* targets PRC2 to hundreds of genes involved in the inhibition of cancer progression (Gupta et al., 2010), and its depletion abrogates the capacity of EZH2 to induce cancer matrix invasion (Gupta et al., 2010). DCGs are enriched in PRC2 and *HOTAIR* target genes, but not in differentially expressed genes. Since a trend of promoter hypermethylation in DCGs exists, it is likely that PRC2 mediates DCG allele-specific long-term repression through DNA methylation-dependent and independent mechanisms.

Another seemingly important factor in DCG events is CTCF, which is a major chromatin “architect.” Notably, CTCF haploinsufficient mice are predisposed to cancer and show and increased variability in CpG methylation genome wide (Kemp et al., 2014). CTCF is involved in imprinting regulation through high-order chromatin loops in their insulator regions (Guibert et al., 2012; Liu et al., 2014). Loss of genomic imprinting (LOI) constitutes a hallmark of many cancers and it is an oncogenic mechanism itself (Holm et al., 2005; Gieni and Hendzel, 2009). Particularly, LOI at the *IGF2* locus involves demethylation of both alleles and depletion of PRC2 and CTCF binding (Li et al., 2014). An important role of CTCF at imprinted genes is to mediate intrachromosomal looping, which permits PRC2 binding and heterochromatin formation (Zhang et al., 2011). Since DCGs are enriched in CTCF peaks and in imprinted genes, it is reasonable to think that local and large chromatin reorganization patterns are involved in the epigenetic regulation of DCGs during carcinogenesis, with a special emphasis on regions of allele specific expression.

Finally, this study uses public retrospective data, and it has limitations and concerns inherent to the study design, the heterogeneity of the platforms and the availability of the data. Five datasets were analyzed, one of which was tumor-normal matched. We observed discordant correlation results in this latter case, which can be related to confounding factors or, more probably, to an epigenetic effect on the non-tumoral adjacent microenvironment. The original study didn't find batch effects due to separate plates or chips, and the methylation levels of normal and cancerous lung samples didn't change substantially between smokers and non-smokers at any locus (Selamat et al., 2012). This dataset is composed of approximately equal parts of smokers and non-smokers, but different proportion of tumor stages (58% Stage I, 19% Stage II, 21% Stage III, and 2% Stage IV), ethnicities and sexes exist. Using *Combat* (Johnson et al., 2007) to adjust the variability associated with these traits lead to exactly the same results. Similarly, we replicated the same observations in the breast cancer cohort after removing the variability associated with age and ethnicity (Pearson's correlations > 0.99; slope ≈ 1), the clearest potential confounders. Although the possible effect of these seems to be minimal, future studies should be carried on data from very homogeneous cohorts in order to validate the formulated hypotheses. For this reason, we propose to develop a new study to corroborate our findings. This proposal would test the correlation changes that exist between cancer tissues and normal-adjacent and non-adjacent ones, along with the chromatin changes accompanying them. The study expects to use sequencing technology instead of arrays in order to inspect events at a base resolution, and it would be accompanied by high-resolution cytogenetic information to detect regions of SCNA. Finally, the proposed study would also try to address the role that CTCF and PRC2 play at DCGs, and the possibility of modulating correlations according to their binding kinetics.

## Conclusion

**Figure 5:**
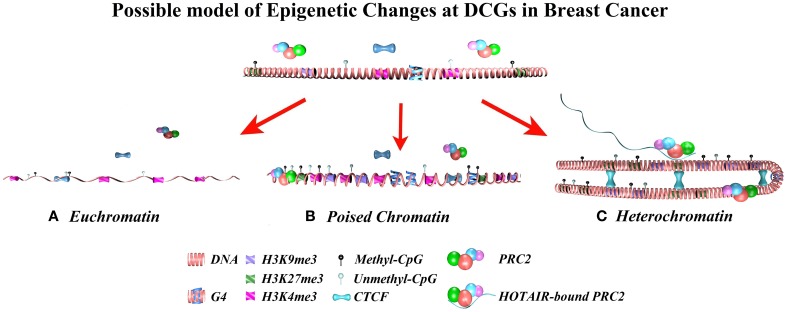
**A model of the differential epigenetic regulation that occurs at DCGs during carcinogenesis is proposed**. This model postulates that PRC2-repressive complexes, CTCF-mediated chromatin loops and G Quadruplex structures cooperate to modulate the chromatin structure toward any of the following three: **(A)** Euchromatin, **(B)** Poised chromatin, or **(C)** Heterochromatin. Note that the potential implication of G4 structures must be interpreted with great caution, since it might be an artifact of the increased GC content found in DCGs. Due to the high enrichment of DCGs in heterochromatin and poised chromatin in model cell lines, these are the most likely states. Indeed, since most of the genes do not show differential expression trends, it is likely that DCGs preferentially indicate bivalent chromatin regions where monoallelic expression switches between cancer and normal cells.

This study provides proof-of-concept for the implication of differential expression-methylation correlation in cancer biology. We have demonstrated general trends of differential expression-methylation in cancer compared with normal tissues, and we identified a group of genes with striking and significant correlation differences in breast cancer, known as DCGs. Breast DCGs were enriched in cell differentiation and cancer-related pathways, with a special emphasis on the family of Homeobox transcription factors. DCGs indicate hotspots of epigenetic reprogramming in cancer, where other epigenetic or genomic factors exceed or modify the effect of DNA methylation. By integrating DCG data with previous epigenomics studies, we discovered that DCGs are markedly enriched in repressive and bivalent chromatin features. Our data supports a model where DCGs undergo epigenetic reprogramming during carcinogenesis triggered by PRC2 redistribution; which involves large and local chromatin reorganization through CTCF (Figure 5). These locations are likely to be prone to chromosomal instability and loss of imprinting, which can be a consequence of epigenetic aberrations in rapidly proliferating cells. Since cancer cells can spontaneously transform into a stem cell-like phenotype modulated by genes within bivalent chromatin regions (Chaffer et al., 2013), it is tempting to speculate that DCGs undergo an epigenetic reprogramming that facilitates monoallelic gene expression and stemness. Moreover, since breast DCGs were neither substantially differentially expressed nor differentially methylated, this suggests that a greater understanding of biological variability can be achieved by integrating expression and methylation data. Thus, it is relevant to corroborate these findings and to expand our knowledge about the mechanisms behind this phenomenon and its functional role in cancer biology.

## Author note

AMO is a young Spanish researcher specialized in cancer genomics. He is not affiliated to any public or private institution and all his research is self-financed with his (scarce) savings. Information requests and comments can be sent to his email or his LinkedIn profile.

## Author contributions

AMO designed the study, carried out all the research and wrote the manuscript.

### Conflict of interest statement

The author declares that the research was conducted in the absence of any commercial or financial relationships that could be construed as a potential conflict of interest.

## Footnotes

^1^Bolstad, M. B. preprocessCore: A Collection of Pre-processing Functions. R package version 1.18.10.

## Supplementary material

Supplementary Figure 1 **Gene Ontology hierarchical diagram that shows significant enrichments of DCGs in lung adenocarcinoma and HNSCC colored in red (5% FDR)**. Scatter plots of expression vs. methylation in cancer and normal samples are reported for the top two genes (for reasons of brevity).

Supplementary Figure 2 **Diagnostic plots of the GAM regression of Z-score Spearman's correlations in normal breast as a function of absolute distance to the closest TSS**. K', EDF, K-index, and *P*-values can be consulted in the top of the image. The plots correspond to *theoretical quantiles* vs. *deviance residuals* (upper-left plot), residuals vs. frequency (lower-left plot), *linear predictor* vs. *residuals* (upper-right plot), and *fitted values* vs. *response* (lower-right plot).

Supplementary Figure 3 **Same as Supplementary Figure 2 for Z-score Speraman's correlations in breast cancer**.

Supplementary Figure 4 **Same as Supplementary Figure 2 but focused on the CpGs falling within a 5000 bp range from the closest TSS**.

Supplementary Figure 5 **Same as Supplementary Figure 3 but focused on the CpGs falling within a 5000 bp range from the closest TSS**.

Supplementary Figure 6 **DCGs can be more positively or negatively correlated in cancer with respect to normal breast tissues**. We performed GAM regression of the correlations in DCGs separately for both groups. Here we report the diagnostic plots of GAM regression in cancer samples of DCGs with higher correlations in breast cancer than in normal breast. Once again, absolute distance to the closest TSS was the independent variable. Plots follow the same order as those listed in Supplementary Figure 2.

Supplementary Figure 7 **DCGs can be more positively or negatively correlated in cancer with respect to normal breast tissues**. We performed GAM regression of the correlations in DCGs separately for both groups. Here we report the diagnostic plots of GAM regression in normal breast samples of DCGs with higher correlations in breast cancer than in normal breast. Once again, absolute distance to the closest TSS was the independent variable. Plots follow the same order as those listed in Supplementary Figure 2.

Supplementary Figure 8 **DCGs can be more positively or negatively correlated in cancer with respect to normal breast tissues**. We performed GAM regression of the correlations in DCGs separately for both groups. Here we report the diagnostic plots of GAM regression in cancer samples of DCGs with higher correlations in normal tissues than in breast cancer. Once again, absolute distance to the closest TSS was the independent variable. Plots follow the same order as those listed in Supplementary Figure 2.

Supplementary Figure 9 **DCGs can be more positively or negatively correlated in cancer with respect to normal breast tissues**. We performed GAM regression of the correlations in DCGs separately for both groups. Here we report the diagnostic plots of GAM regression in normal samples of DCGs with higher correlations in normal tissues than in breast cancer. Once again, absolute distance to the closest TSS was the independent variable. Plots follow the same order as those listed in Supplementary Figure 2.

Supplementary Figure 10 **(A)** DCG promoters' differential methylation results. Average promoter methylation was calculated for each DCG and differential methylation was studied using the SAM algorithm. The black dots indicate differences in methylation values between cancer and normal, and the red dots indicate genes whose promoters are significantly hypermethylated in cancer compared with normal breast (5% FDR). Note the clear tendency for almost all promoters to be more methylated in cancer than in normal breast (black dots above the expected black line). **(B)** DCG non-promoter regions' differential methylation results. The differences between cancer and normal breast samples are less obvious than in the previous case, but a group of DCGs are hypermethylated in cancer at a 5% FDR (red dots). Note that in this case, DCGs do not show a general tendency to be hypermethylated in cancer. **(C)** SAM results for DCGs' differential expression. Note that although most genes do not show differential expression patterns at a 5% FDR, a small group is down-regulated in cancer (green dots).

Supplementary Figure 11 **Gene Ontology hierarchical diagram that shows significant enrichments of DCGs in breast cancer colored in red (5% FDR)**.

Supplementary Table 1 **DCG list in HNSCC and Lung Adenocarcinoma**. The list of all the genes included in the analysis is also reported.

Supplementary Table 2 **Lists of DCGs in breast cancer**. Two lists are reported since Stouffer's test must be performed on one-sided *P*-values.

Supplementary Table 3 **Enrichment of breast DCGs in *InterPro* domains**. Data was analyzed in *GeneCodis*.

Supplementary Table 4 **Enrichment of breast DCGs in chromosomal regions**. Data was analyzed in *Web Gestalt*.

Supplementary Table 5 **Enrichment of breast DCGs in pathways of the *Pathways Commons* database**. Data was analyzed in Web Gestalt.

Supplementary Table 6 **Enrichment of breast DCGs in conserved TFBS**. Data was analyzed in *GeneCodis*.

Supplementary Table 7 **Enrichment of breast DCGs-related miRNA target genes in *KEGG* pathways**. Data was analyzed in Diana Tools.

Supplementary Table 8 **Enrichment of breast DCGs in (1) TF peaks in three difference cell lines; (2) Meta-analysis of the TF tracks present in all three cell lines; (3) chromatin marks across four model cell lines; and (4) chromatin state segmentation across four model cell lines**.

## Abbreviations

DCG: Differentially Correlated Gene
cDMR: cancer-specific Differentially Methylated Region
TSS: Transcription Start Site
PMD: Partially Methylated Domain
HMD: Heavily Methylated Domain
PRC2: Polycomb Repressive Complex 2
LAD: Lamina-Associated Domain
EI: Euchromatin Island
SCNA: Somatic Copy Number Aberration
TF: Transcription Factor
TFBS: Transcription Factor Binding Site
bp: base pair
LOI: Loss of Genomic Imprinting
HNSCC: Head and Neck Squamous Cell Carcinoma
SAM: Significance Analysis for Microarrays
FDR: False Discovery Rate
Wilcox: Wilcoxon Rank Sum Test.

## References

[B1] Abelson P. R. (1995). Statistics as Principled Argument. New York, NY: Taylor & Francis Group.

[B2] Agarwal T. Roy S. Kumar S. Chakraborty T. K. Maiti S. (2014). In the sense of transcription regulation by G-quadruplexes: asymmetric effects in sense and antisense strands. Biochemistry 53, 3711–3718. 10.1021/bi401451q 24850370

[B3] Ashburner M. Ball C. A. Blake J. A. Botstein D. Butler H. Cherry J. M.. (2000). Gene ontology: tool for the unification of biology. The Gene Ontology Consortium. Nat. Genet. 25, 25–29. 10.1038/75556 10802651 PMC3037419

[B4] Bae W. K. Yoo K. H. Lee J. S. Kim Y. Chung I. J. Park M. H. (2014). The methyltransferase EZH2 is not required for mammary cancer development, although high EZH2 and low H3K27me3 correlate with poor prognosis of ER-positive breast cancers. Mol. Carcinog. 10.1002/mc.22188. [Epub ahead of print]. PMC4286524 25043748

[B5] Barrett T. Wilhite S. E. Ledoux P. Evangelista C. Kim I. F. Tomashevsky M.. (2013). NCBI GEO: archive for functional genomics data sets—update. Nucleic Acids Res. 41, D991–D995. 10.1093/nar/gks1193 23193258 PMC3531084

[B6] Bell J. T. Pai A. A. Pickrell J. K. Gaffney D. J. Pique-Regi R. Degner J. F.. (2011). DNA methylation patterns associate with genetic and gene expression variation in HapMap cell lines. Genome Biol. 12, R10. 10.1186/gb-2011-12-1-r10 21251332 PMC3091299

[B7] Berman B. P. Weisenberger D. J. Aman J. F. Hinoue T. Ramjan Z. Liu Y.. (2011). Regions of focal DNA hypermethylation and long-range hypomethylation in colorectal cancer coincide with nuclear lamina-associated domains. Nat. Genet. 44, 40–46. 10.1038/ng.969 22120008 PMC4309644

[B8] Beroukhim R. Mermel C. H. Porter D. Wei G. Raychaudhuri S. Donovan J.. (2010). The landscape of somatic copy-number alteration across human cancers. Nature 463, 899–905. 10.1038/nature08822 20164920 PMC2826709

[B9] Blumenthal R. D. Hansen H. J. Goldenberg D. M. (2005). Inhibition of adhesion, invasion, and metastasis by antibodies targeting CEACAM6 (NCA-90) and CEACAM5 (Carcinoembryonic Antigen). Cancer Res. 65, 8809–8817. 10.1158/0008-5472.CAN-05-0420 16204051

[B11] Bork S. Pfister S. Witt H. Horn P. Korn B. Ho A. D.. (2010). DNA methylation pattern changes upon long-term culture and aging of human mesenchymal stromal cells. Aging Cell 9, 54–63. 10.1111/j.1474-9726.2009.00535.x 19895632 PMC2814091

[B12] Boros J. Arnoult N. Stroobant V. Collet J. F. Decottignies A. (2014). Polycomb Repressive Complex 2 and H3K27me3 cooperate with H3K9 methylation to maintain Heterochromatin Protein 1α at chromatin. Mol. Cell. Biol. 34, 3662–3674. 10.1128/MCB.00205-14 25047840 PMC4187721

[B13] Brenet F. Moh M. Funk P. Feierstein E. Viale A. J. Socci N. D.. (2011). DNA methylation of the first exon is tightly linked to transcriptional silencing. PLoS ONE 6:e14524. 10.1371/journal.pone.0014524 21267076 PMC3022582

[B14] Cerami E. G. Gross B. E. Demir E. Rodchenkov I. Babur O. Anwar N.. (2011). Pathway Commons, a web resource for biological pathway data. Nucleic Acids Res. 39, D685–D690. 10.1093/nar/gkq1039 21071392 PMC3013659

[B15] Chaffer C. L. Marjanovic N. D. Lee T. Bell G. Kleer C. G. Reinhardt F.. (2013). Poised chromatin at the ZEB1 promoter enables breast cancer cell plasticity and enhances tumorigenicity. Cell 154, 61–74. 10.1016/j.cell.2013.06.005 23827675 PMC4015106

[B16] Creelan B. C. Antonia S. Noyes D. Hunter T. B. Simon G. R. Bepler G.. (2013). Phase II trial of a GM-CSF-producing and CD40L-expressing bystander cell line combined with an allogeneic tumor cell-based vaccine for refractory lung adenocarcinoma. J. Immunother. 36, 442–450. 10.1097/CJI.0b013e3182a80237 23994887 PMC3846277

[B18] Doi A. Park I. H. Wen B. Murakami P. Aryee M. J. Irizarry R.. (2009). Differential methylation of tissue- and cancer-specific CpG island shores distinguishes human induced pluripotent stem cells, embryonic stem cells and fibroblasts. Nat. Genet. 41, 1350–1353. 10.1038/ng.471 19881528 PMC2958040

[B19] Durinck S. Spellman T. P. Birney E. Huber W. (2009). Mapping identifiers for the integration of genomic datasets with the R/Bioconductor package biomaRt. Nat. Protoc. 4, 1184–1191. 10.1038/nprot.2009.97 19617889 PMC3159387

[B20] ENCODE Project Consortium. (2012). An integrated encyclopedia of DNA elements in the human genome. Nature 489, 57–74 10.1038/nature11247 22955616 PMC3439153

[B21] Fertig E. J. Markovic A. Danilova L. V. Gaykalova D. A. Cope L. Chung C. H.. (2013). Preferential activation of the hedgehog pathway by epigenetic modulations in HPV negative HNSCC identified with meta-pathway analysis. PLoS ONE 8:e78127. 10.1371/journal.pone.0078127 24223768 PMC3817178

[B22] Ghavifekr F. M. Farshdousti H. M. Shanehbandi D. Baradaran B. (2014). DNA methylation pattern as important epigenetic criterion in cancer. Genet. Res. Int. 2013:317569. 10.1155/2013/317569 24455281 PMC3884803

[B23] Gieni R. S. Hendzel M. J. (2009). Polycomb group protein gene silencing, non-coding RNA, stem cells, and cancer. Biochem. Cell Biol. 87, 711–746. 10.1139/O09-057 19898523

[B24] Gonzalez M. E. Moore H. M. Li X. Toy K. A. Huang W. Sabel M. S.. (2014). EZH2 expands breast stem cells through activation of NOTCH1 signaling. Proc. Natl. Acad. Sci. U.S.A. 111, 3098–3103. 10.1073/pnas.1308953111 24516139 PMC3939892

[B25] Goodwin D. L. Leech L. L. (2006). Understanding correlation. Factors that affect the size of r. J. Exp. Educ. 74, 251–266. 10.3200/JEXE.74.3.249-26611967674

[B26] Govindan S. V. Cardillo T. M. Moon S. J. Hansen H. J. Goldenberg D. M. (2009). CEACAM5-targeted therapy of human colonic and pancreatic cancer xenografts with potent labetuzumab-SN-38 immunoconjugates. Clin. Cancer Res. 15, 6052–6061. 10.1158/1078-0432.CCR-09-0586 19789330 PMC2769088

[B27] Guibert S. Zhao Z. Sjölinder M. Göndör A. Fernandez A. Pant V.. (2012). CTCF-binding sites within the H19 ICR differentially regulate local chromatin structures and cis-acting functions. Epigenetics 7, 361–369. 10.4161/epi.19487 22415163

[B28] Guillaumet-Adkins A. Richter J. Odero M. D. Sandoval J. Agirre X. Catala A.. (2014). Hypermethylation of the alternative AWT1 promoter in hematological malignancies is a highly specific marker for acute myeloid leukemias despite high expression levels. J. Hematol. Oncol. 7, 4. 10.1186/1756-8722-7-4 24405639 PMC3900738

[B29] Gupta R. A. Shah N. Wang K. C. Kim J. Horlings H. M. Wong D. J.. (2010). Long non-coding RNA HOTAIR reprograms chromatin state to promote cancer metastasis. Nature 464, 1071–1076. 10.1038/nature08975 20393566 PMC3049919

[B30] Halder R. Halder K. Sharma P. Garg G. Sengupta S. Chowdhury S. (2010). Guanine quadruplex DNA structure restricts methylation of CpG dinucleotides genome-wide. Mol. Biosyst. 6, 2439–2447. 10.1039/c0mb00009d 20877913

[B31] Han H. Cortez C. C. Yang X. Nichols P. W. Jones P. A. Liang G. (2011). DNA methylation directly silences genes with non-CpG island promoters and establishes a nucleosome occupied promoter. Hum. Mol. Genet. 20, 4299–4310. 10.1093/hmg/ddr356 21835883 PMC3196883

[B32] Hansen K. D. Timp W. Bravo H. C. Sabunciyan S. Langmead B. McDonald O. G.. (2011). Increased methylation variation in epigenetic domains across cancer types. Nat. Genet. 43, 768-75. 10.1038/ng.865 21706001 PMC3145050

[B33] Holm T. M. Jackson-Grusby L. Brambrink T. Yamada Y. Rideout W. M.. (2005). Global loss of imprinting leads to widespread tumorigenesis in adult mice. Cancer Cell 8, 275–285. 10.1016/j.ccr.2005.09.007 16226703

[B34] Hon G. C. Hawkins R. D. Caballero O. L. Lo C. Lister R. Pelizzola M.. (2012). Global DNA hypomethylation coupled to repressive chromatin domain formation and gene silencing in breast cancer. Genome Res. 2, 246–258. 10.1101/gr.125872.111 22156296 PMC3266032

[B35] Houseman E. A. Christensen B. C. Karagas M. R. Wrensch M. R. Nelson H. H. Wiemels J. L.. (2009). Copy number variation has little impact on bead-array-based measures of DNA methylation. Bioinformatics 25, 1999–2005. 10.1093/bioinformatics/btp364 19542153 PMC2723008

[B36] Huang T. H. Yeh P. L. Martin M. B. Straub R. E. Gilliam T. C. Caldwell C. W.. (1995). Genetic alterations of microsatellites on chromosome 18 in human breast carcinoma. Diagn. Mol. Pathol. 4, 66–72. 10.1097/00019606-199503000-00012 7735559

[B37] Huppert J. L. Balasubramanian S. (2007). G-quadruplexes in promoters throughout the human genome. Nucleic Acids Res. 35, 406–413. 10.1093/nar/gkl1057 17169996 PMC1802602

[B38] Jirtle L. J. (1997). Geneimprint. Geneimprint database. Available online at: http://www.geneimprint.com/site/genes-by-species

[B39] Jjingo D. Conley A. B. Yi S. V. Lunyak V. V. Jordan I. K. (2012). On the presence and role of human gene-body DNA methylation. Oncotarget 3, 462–474. 22577155 10.18632/oncotarget.497 PMC3380580

[B40] Johnson W. E. Li C. Rabinovic A. (2007). Adjusting batch effects in microarray expression data using empirical Bayes methods. Biostatistics 8, 118–127. 10.1093/biostatistics/kxj037 16632515

[B41] Jones P. Binns D. Chang H. Y. Fraser M. Li W. McAnulla C.. (2014). InterProScan 5: genome-scale protein function classification. Bioinformatics 30, 1236–1240. 10.1093/bioinformatics/btu031 24451626 PMC3998142

[B42] Kanduri M. Sander B. Ntoufa S. Papakonstantinou N. Sutton L. A. Stamatopoulos K.. (2013). A key role for EZH2 in epigenetic silencing of HOX genes in mantle cell lymphoma. Epigenetics 8, 1280–1288. 10.4161/epi.26546 24107828 PMC3933489

[B43] Kanehisa M. (2013). Molecular network analysis of diseases and drugs in KEGG. Methods Mol. Biol. 939, 263–275. 10.1007/978-1-62703-107-3_17 23192552

[B44] Kemp C. J. Moore J. M. Moser R. Bernard B. Teater M. Smith L. E.. (2014). CTCF haploinsufficiency destabilizes DNA methylation and predisposes to cancer. Cell Rep. 7, 1020–1029. 10.1016/j.celrep.2014.04.004 24794443 PMC4040130

[B45] Kim Y. J. Yoon H. Y. Kim J. S. Kang H. W. Min B. D. Kim S. K.. (2013). HOXA9, ISL1 and ALDH1A3 methylation patterns as prognostic markers for nonmuscle invasive bladder cancer: array-based DNA methylation and expression profiling. Int. J. Cancer 133, 1135–1142. 10.1002/ijc.28121 23436614

[B46] Klipp E. (2009). Systems Biology: A Textbook. Weinheim: Wiley-VCH.

[B47] Kresse S. H. Rydbeck H. Skårn M. Namløs H. M. Barragan-Polania A. H. Cleton-Jansen A. M.. (2012). Integrative analysis reveals relationships of genetic and epigenetic alterations in osteosarcoma. PLoS ONE 7:e48262. 10.1371/journal.pone.0048262 23144859 PMC3492335

[B48] Li T. Chen H. Li W. Cui J. Wang G. Hu X.. (2014). Promoter histone H3K27 methylation in the control of IGF2 imprinting in human tumor cell lines. Hum. Mol. Genet. 23, 117–128. 10.1093/hmg/ddt405 23962719 PMC3857949

[B49] Liu Q. Yang B. Xie X. Wei L. Liu W. Yang W.. (2014). Vigilin interacts with CCCTC-binding factor (CTCF) and is involved in CTCF-dependent regulation of the imprinted genes Igf2 and H19. FEBS J. 281, 2713–2725. 10.1111/febs.12816 24725430

[B50] Liu Z. G. Lei Y. Y. Li W. W. Chen Z. G. (2013). The co-expression of ERβ2 and IL-12Rβ2 is better prognostic factor in non-small-cell lung cancer progression. Med. Oncol. 30, 592. 10.1007/s12032-013-0592-x 23677568

[B51] Lou S. Lee H. M. Qin H. Li J. W. Gao Z. Liu X.. (2014). Whole-genome bisulfite sequencing of multiple individuals reveals complementary roles of promoter and gene body methylation in transcriptional regulation. Genome Biol. 15, 408. 10.1186/s13059-014-0408-0 25074712 PMC4189148

[B52] McEntyre J. Lipman D. (2001). PubMed: bridging the information gap. CMAJ 164, 1317–1319. 11341144 PMC81025

[B53] Moran A. E. Kovacsovics-Bankowski M. Weinberg A. D. (2013). The TNFRs OX40, 4-1BB, and CD40 as targets for cancer immunotherapy. Curr. Opin. Immunol. 25, 230–237. 10.1016/j.coi.2013.01.004 23414607 PMC3815601

[B54] Morey L. Helin K. (2010). Polycomb group protein-mediated repression of transcription. Trends Biochem. Sci. 35, 323–332. 10.1016/j.tibs.2010.02.009 20346678

[B55a] Mudholkar G. S. (2006). Fisher's Z-Transformation, in Encyclopedia of Statistical Sciences, eds Kotz S. Read C. B. Balakrishnan N. Vidakovic B. (New York, NY: Wiley Online).

[B55] Myers L. Sirois M. J. (2006). Differences between Spearman Correlation Coefficients, in Encyclopedia of Statistical Sciences (John Wiley & Sons, Inc.), 12.

[B56] R Core Team (2012). R: A Language and Environment for Statistical Computing. R Foundation for Statistical Computing. Avaliable online at: http://www.R-project.org/

[B57] Rakhmilevich A. L. Alderson K. L. Sondel P. M. (2012). T-cell-independent antitumor effects of CD40 ligation. Int. Rev. Immunol. 31, 267–278. 10.3109/08830185.2012.698337 22804571 PMC3537496

[B58] Sandoval J. Heyn H. Moran S. Serra-Musach J. Pujana M. A. Bibikova M.. (2011). Validation of a DNA methylation microarray for 450,000 CpG sites in the human genome. Epigenetics 6, 692–702. 10.4161/epi.6.6.16196 21593595

[B59] Sandve G. K. Gundersen S. Rydbeck H. Glad I. K. Holden L. Holden M.. (2010). The Genomic HyperBrowser: inferential genomics at the sequence level. Genome Biol. 11, R121. 10.1186/gb-2010-11-12-r121 21182759 PMC3046481

[B60] Saxonov S. Berg P. Brutlag D. L. (2006). A genome-wide analysis of CpG dinucleotides in the human genome distinguishes two distinct classes of promoters. Proc. Natl. Acad. Sci. U.S.A. 103, 1412–1417. 10.1073/pnas.0510310103 16432200 PMC1345710

[B61] Schroeder D. I. Lott P. Korf I. LaSalle J. M. (2011). Large-scale methylation domains mark a functional subset of neuronally expressed genes. Genome Res. 21, 1583–1591. 10.1101/gr.119131.110 21784875 PMC3202276

[B62] Selamat S. A. Chung B. S. Girard L. Zhang W. Zhang Y. Campan M.. (2012). Genome-scale analysis of DNA methylation in lung adenocarcinoma and integration with mRNA expression. Genome Res. 22, 1197–1211. 10.1101/gr.132662.111 22613842 PMC3396362

[B63] Shann Y. J. Cheng C. Chiao C. H. Chen D. T. Li P. H. Hsu M. T. (2008). Genome-wide mapping and characterization of hypomethylated sites in human tissues and breast cancer cell lines. Genome Res. 18, 791–801. 10.1101/gr.070961.107 18256232 PMC2336806

[B64] Sørensen K. P. Thomassen M. Tan Q. Bak M. Cold S. Burton M.. (2013). Long non-coding RNA HOTAIR is an independent prognostic marker of metastasis in estrogen receptor-positive primary breast cancer. Breast Cancer Res. Treat. 142, 529–536. 10.1007/s10549-013-2776-7 24258260

[B65] Suzuki K. Kadota K. Sima C. S. Nitadori J. Rusch V. W. Travis W. D.. (2013). Clinical impact of immune microenvironment in stage I lung adenocarcinoma: tumor interleukin-12 receptor β2 (IL-12Rβ2), IL-7R, and stromal FoxP3/CD3 ratio are independent predictors of recurrence. J. Clin. Oncol. 31, 490–498. 10.1200/JCO.2012.45.2052 23269987 PMC3731922

[B66] Szulwach K. E. Jin P. (2014). Integrating DNA methylation dynamics into a framework for understanding epigenetic codes. Bioessays 36, 107–117. 10.1002/bies.201300090 24242211 PMC4278575

[B67] Tabas-Madrid D. Nogales-Cadenas R. Pascual-Montano A. (2012). GeneCodis3: a non-redundant and modular enrichment analysis tool for functional genomics. Nucleic Acids Res. 40, W478–W483. 10.1093/nar/gks402 22573175 PMC3394297

[B68] Terunuma A. Putluri N. Mishra P. Mathé E. A. Dorsey T. H. Yi M.. (2014). MYC-driven accumulation of 2-hydroxyglutarate is associated with breast cancer prognosis. J. Clin. Invest. 124, 398–412. 10.1172/JCI71180 24316975 PMC3871244

[B69] Tusher V. G. Tibshirani R. Chu G. (2001). Significance analysis of microarrays applied to the ionizing radiation response. Proc. Natl. Acad. Sci. U.S.A. 98, 5116–5121. 10.1073/pnas.091062498 11309499 PMC33173

[B70] Urosevic J. Garcia-Albéniz X. Planet E. Real S. Céspedes M. V. Guiu M.. (2014). Colon cancer cells colonize the lung from established liver metastases through p38 MAPK signalling and PTHLH. Nat. Cell Biol. 16, 685–694. 10.1038/ncb2977 24880666

[B71] Vanderkraats N. D. Hiken J. F. Decker K. F. Edwards J. R. (2013). Discovering high-resolution patterns of differential DNA methylation that correlate with gene expression changes. Nucleic Acids Res. 41, 6816–6827. 10.1093/nar/gkt482 23748561 PMC3737560

[B72] Vlachos I. S. Kostoulas N. Vergoulis T. Georgakilas G. Reczko M. Maragkakis M.. (2012). DIANA miRPath v.2.0: investigating the combinatorial effect of microRNAs in pathways. Nucleic Acids Res. 40, W498–W504. 10.1093/nar/gks494 22649059 PMC3394305

[B73] Wagner J. R. Busche S. Ge B. Kwan T. Pastinen T. Blanchette M. (2014). The relationship between DNA methylation, genetic and expression inter-individual variation in untransformed human fibroblasts. Genome Biol. 15, R37. 10.1186/gb-2014-15-2-r37 24555846 PMC4053980

[B74] Wang J. Duncan D. Shi Z. Zhang B. (2013). WEB-based GEne SeT AnaLysis Toolkit (WebGestalt): update 2013. Nucleic Acids Res. 41, W77–W83. 10.1093/nar/gkt439 23703215 PMC3692109

[B75] Wang Y. Meng L. Hu H. Zhang Y. Zhao C. Li Q.. (2011). Oct-4B isoform is differentially expressed in breast cancer cells: hypermethylation of regulatory elements of Oct-4A suggests an alternative promoter and transcriptional start site for Oct-4B transcription. Biosci. Rep. 31, 109–115. 10.1042/BSR20100033 20433421

[B76] Wen B. Wu H. Loh Y. H. Briem E. Daley G. Q. Feinberg A. P. (2012). Euchromatin islands in large heterochromatin domains are enriched for CTCF binding and differentially DNA-methylated regions. BMC Genomics 13:566. 10.1186/1471-2164-13-566 23102236 PMC3507770

[B77] Wickham H. (2009). ggplot2: Elegant Graphics for Data Analysis. New York, NY: Springer.

[B78] Wood S. N. (2011). Fast stable restricted maximum likelihood and marginal likelihood estimation of semiparametric generalized linear models. J. R. Stat. Soc. 73, 3–36 10.1111/j.1467-9868.2010.00749.x

[B79] Wu X. Gong Y. Yue J. Qiang B. Yuan J. Peng X. (2008). Cooperation between EZH2, NSPc1-mediated histone H2A ubiquitination and Dnmt1 in HOX gene silencing. Nucleic Acids Res. 36, 3590–3599. 10.1093/nar/gkn243 18460542 PMC2441805

[B80] Yu W. Kanaan Y. Bae Y. K. Gabrielson E. (2009). Chromosomal changes in aggressive breast cancers with basal-like features. Cancer Genet. Cytogenet. 193, 29–37. 10.1016/j.cancergencyto.2009.03.017 19602461 PMC2768045

[B81] Zhang H. Niu B. Hu J. F. Ge S. Wang H. Li T.. (2011). Interruption of intrachromosomal looping by CCCTC binding factor decoy proteins abrogates genomic imprinting of human insulin-like growth factor II. J. Cell Biol. 193, 475–487. 10.1083/jcb.201101021 21536749 PMC3087012

[B82] Zheng C. Feng J. Lu D. Wang P. Xing S. Coll J. L.. (2011). A novel anti-CEACAM5 monoclonal antibody, CC4, suppresses colorectal tumor growth and enhances NK cells-mediated tumor immunity. PLoS ONE 6:e21146. 10.1371/journal.pone.0021146 21731662 PMC3120848

