# Supplementary figures and images for: Hidden among the crowd: differential DNA methylation-expression correlations in cancer occur at important oncogenic pathways
Source: Front Genet. 2015 May 13;6:163. doi: 10.3389/fgene.2015.00163 (PMC4429616; doi:10.3389/fgene.2015.00163)

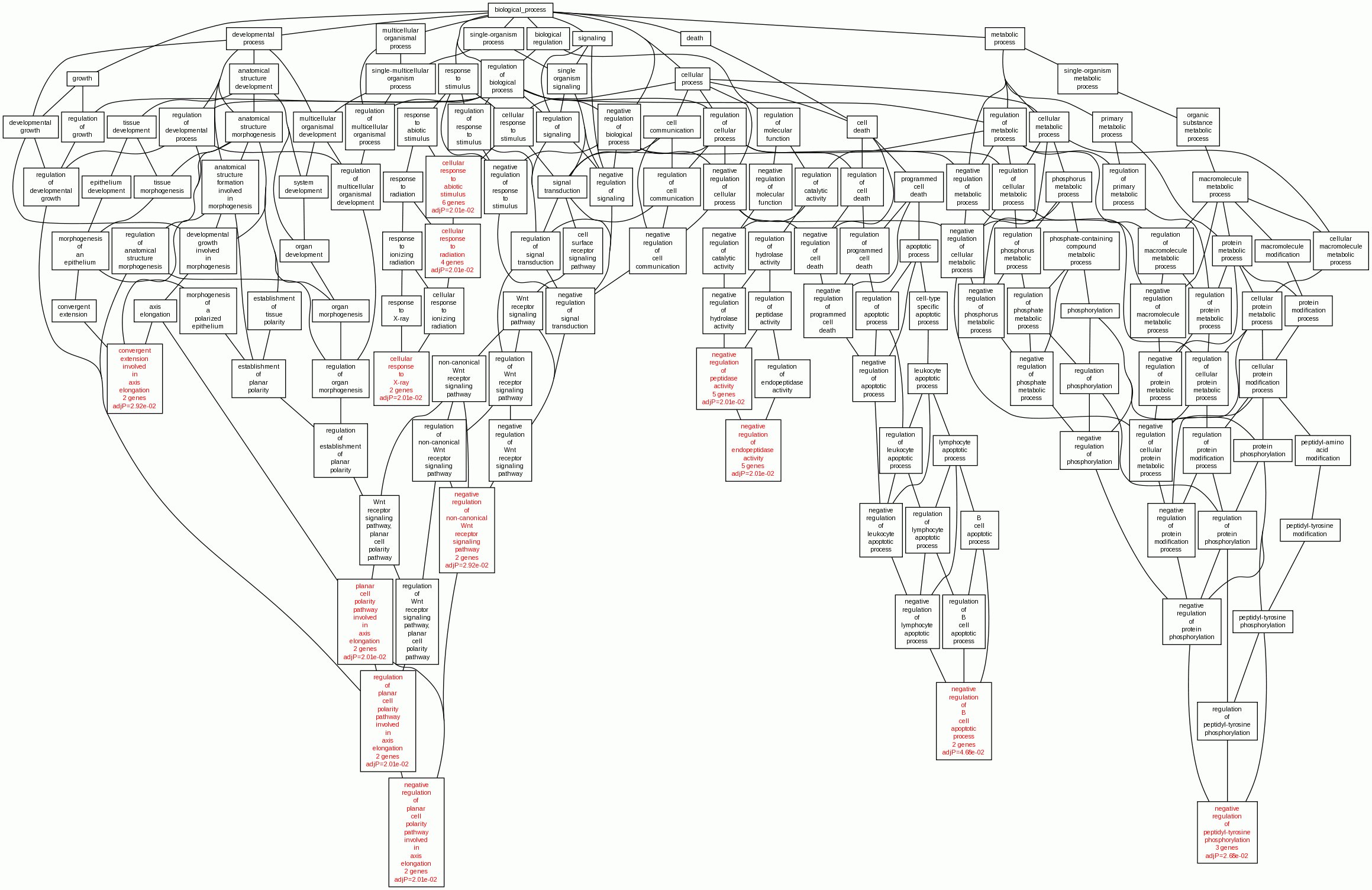

Supplement: Supplementary Figure 1 — Gene Ontology hierarchical diagram that shows significant enrichments of DCGs in lung adenocarcinoma and HNSCC colored in red (5% FDR). Scatter plots of expression vs. methylation in cancer and normal samples are reported for the top two genes (for reasons of brevity). [file Presentation1.ZIP › Supplementary Figure 1.jpg]

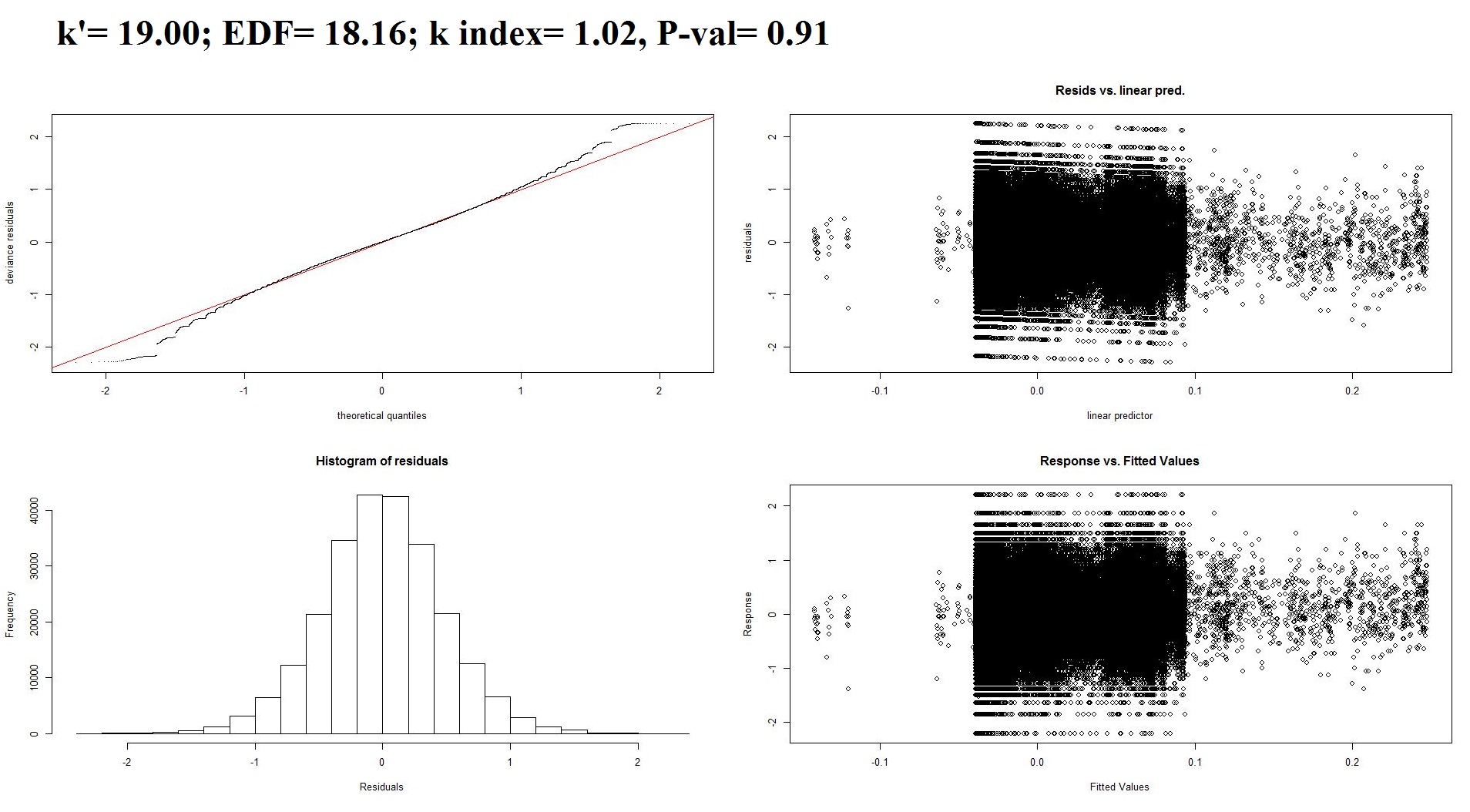

Supplement: Supplementary Figure 1 — Gene Ontology hierarchical diagram that shows significant enrichments of DCGs in lung adenocarcinoma and HNSCC colored in red (5% FDR). Scatter plots of expression vs. methylation in cancer and normal samples are reported for the top two genes (for reasons of brevity). [file Presentation1.ZIP › Supplementary Figure 2.JPG]

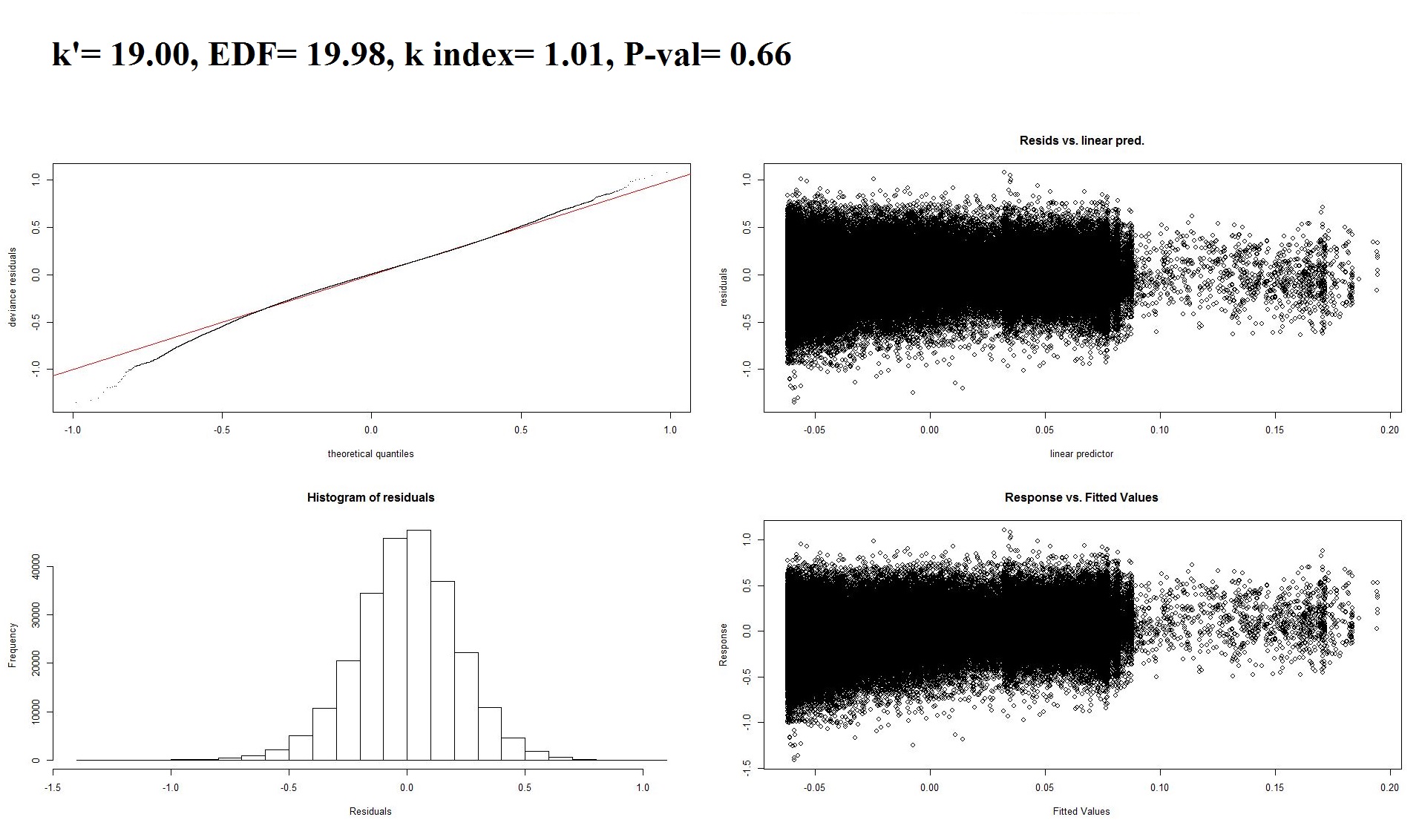

Supplement: Supplementary Figure 1 — Gene Ontology hierarchical diagram that shows significant enrichments of DCGs in lung adenocarcinoma and HNSCC colored in red (5% FDR). Scatter plots of expression vs. methylation in cancer and normal samples are reported for the top two genes (for reasons of brevity). [file Presentation1.ZIP › Supplementary Figure 3.JPG]

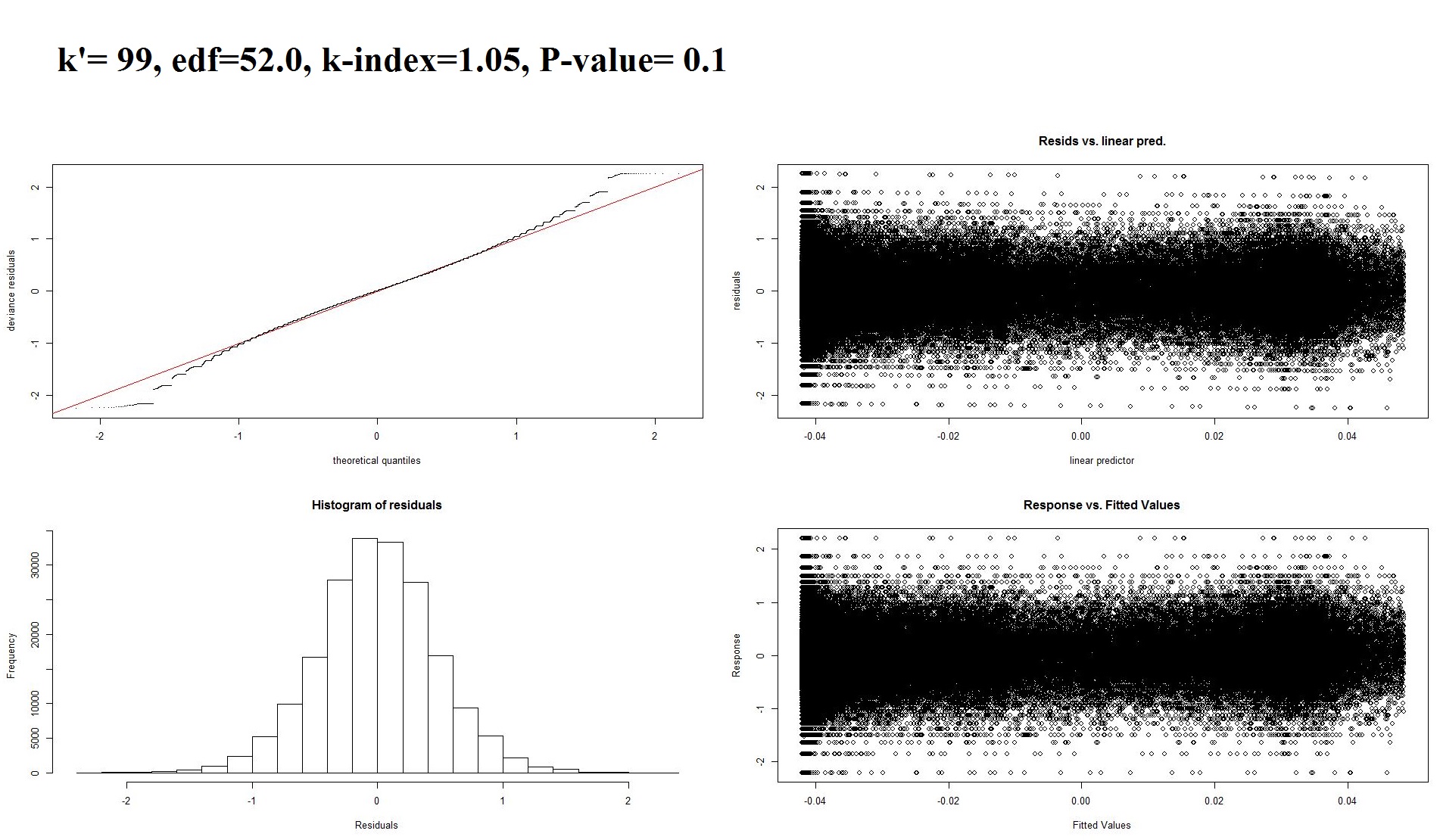

Supplement: Supplementary Figure 1 — Gene Ontology hierarchical diagram that shows significant enrichments of DCGs in lung adenocarcinoma and HNSCC colored in red (5% FDR). Scatter plots of expression vs. methylation in cancer and normal samples are reported for the top two genes (for reasons of brevity). [file Presentation1.ZIP › Supplementary Figure 4.JPG]

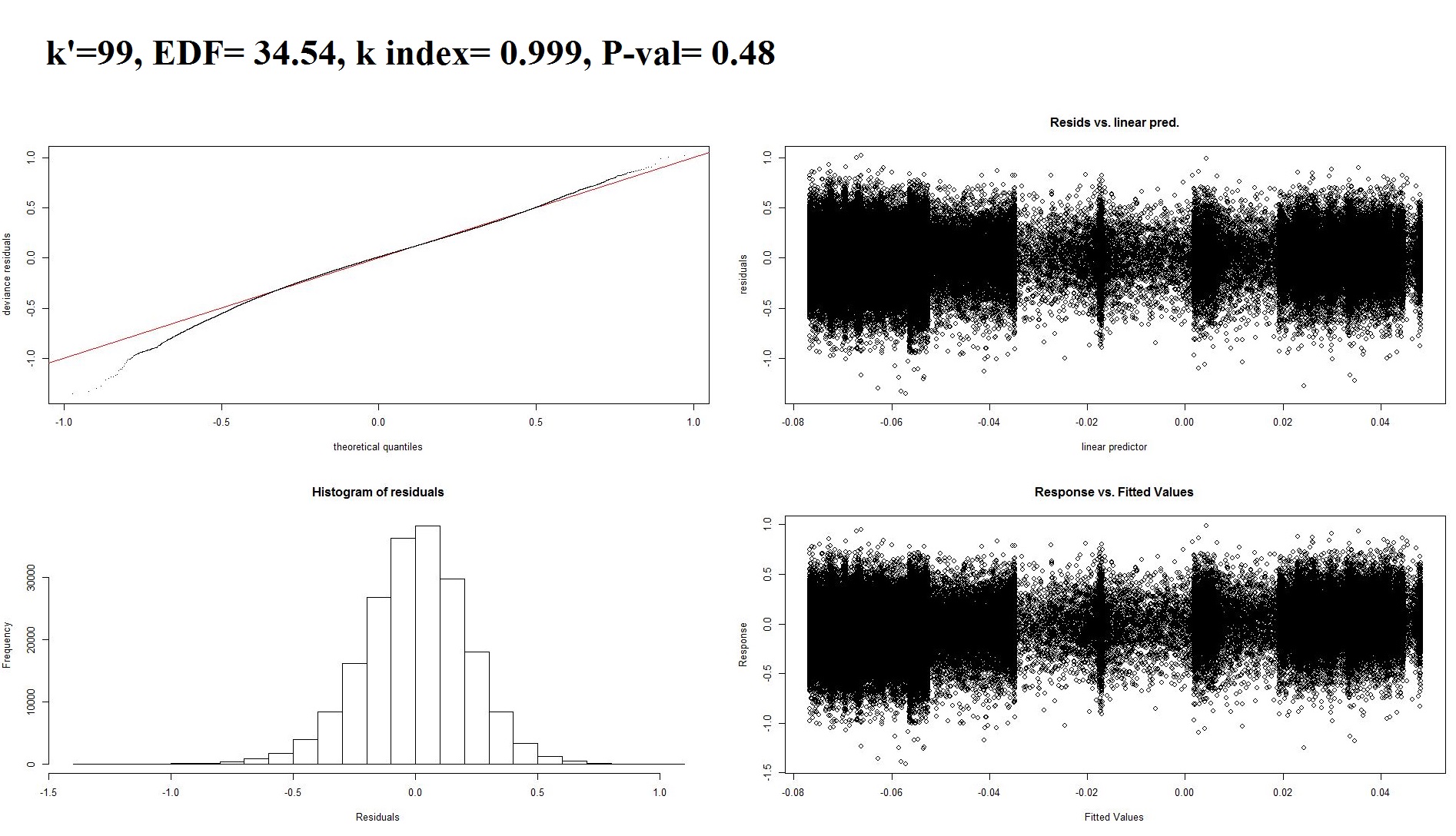

Supplement: Supplementary Figure 1 — Gene Ontology hierarchical diagram that shows significant enrichments of DCGs in lung adenocarcinoma and HNSCC colored in red (5% FDR). Scatter plots of expression vs. methylation in cancer and normal samples are reported for the top two genes (for reasons of brevity). [file Presentation1.ZIP › Supplementary Figure 5.JPG]

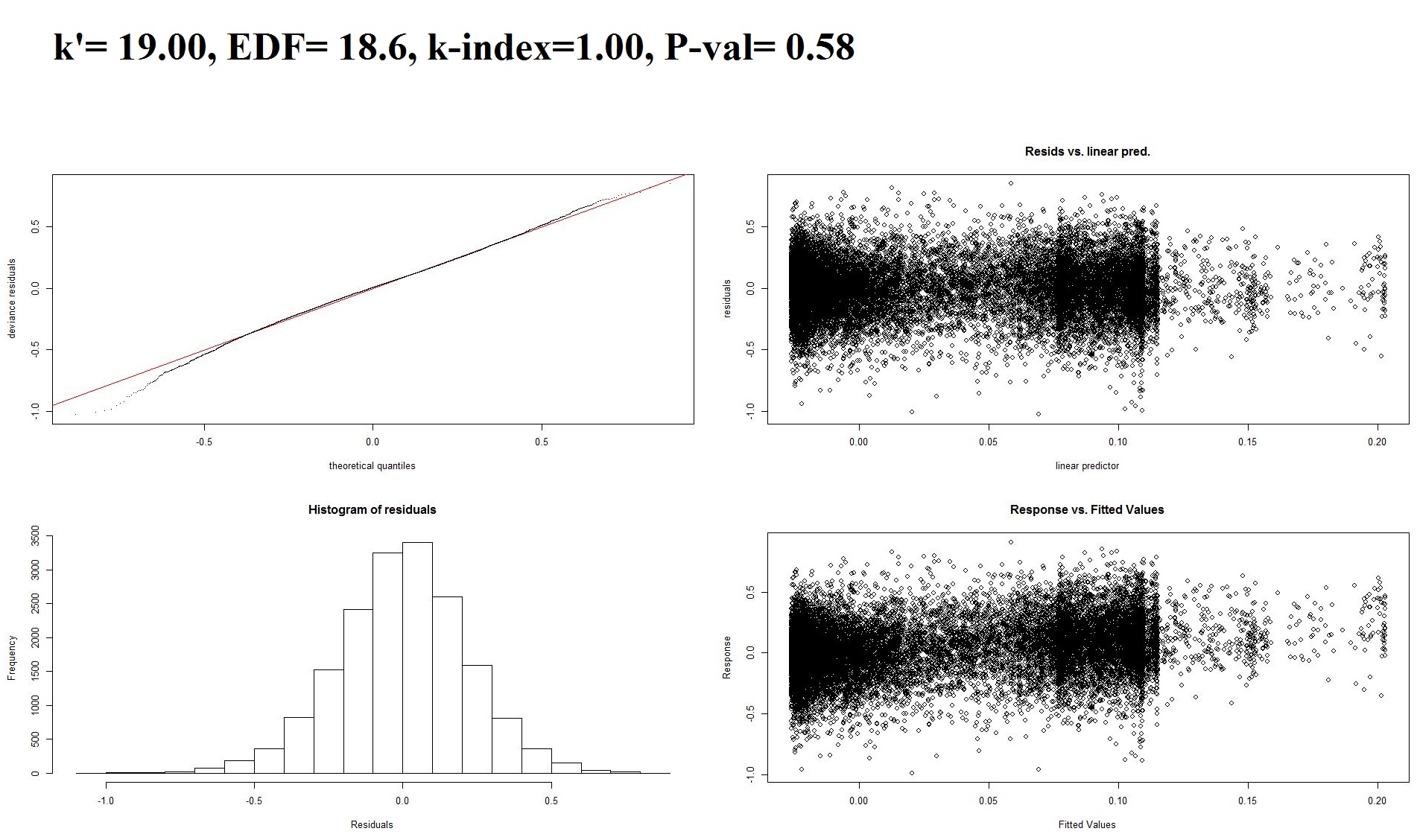

Supplement: Supplementary Figure 1 — Gene Ontology hierarchical diagram that shows significant enrichments of DCGs in lung adenocarcinoma and HNSCC colored in red (5% FDR). Scatter plots of expression vs. methylation in cancer and normal samples are reported for the top two genes (for reasons of brevity). [file Presentation1.ZIP › Supplementary Figure 6.JPG]

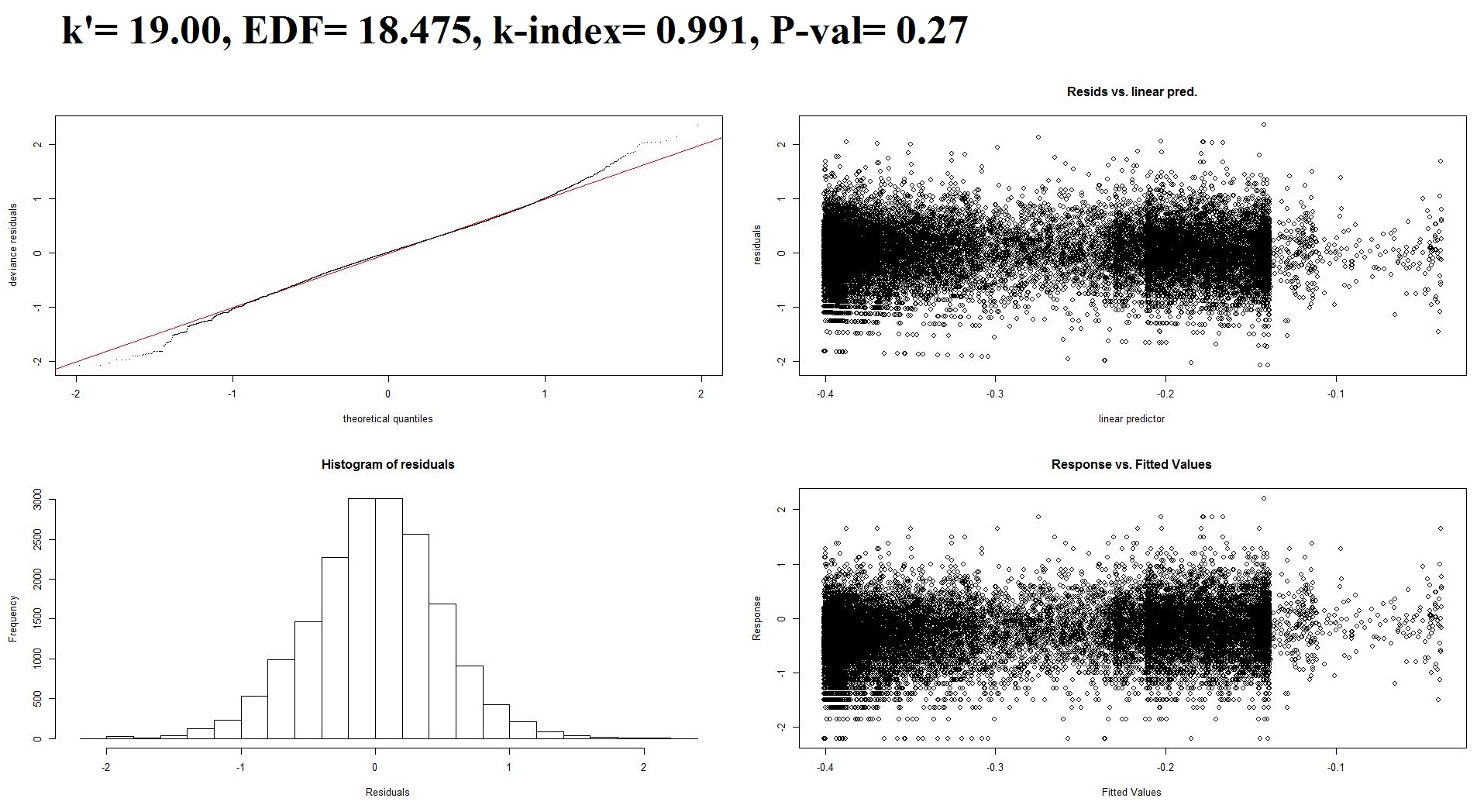

Supplement: Supplementary Figure 1 — Gene Ontology hierarchical diagram that shows significant enrichments of DCGs in lung adenocarcinoma and HNSCC colored in red (5% FDR). Scatter plots of expression vs. methylation in cancer and normal samples are reported for the top two genes (for reasons of brevity). [file Presentation1.ZIP › Supplementary Figure 7.JPG]

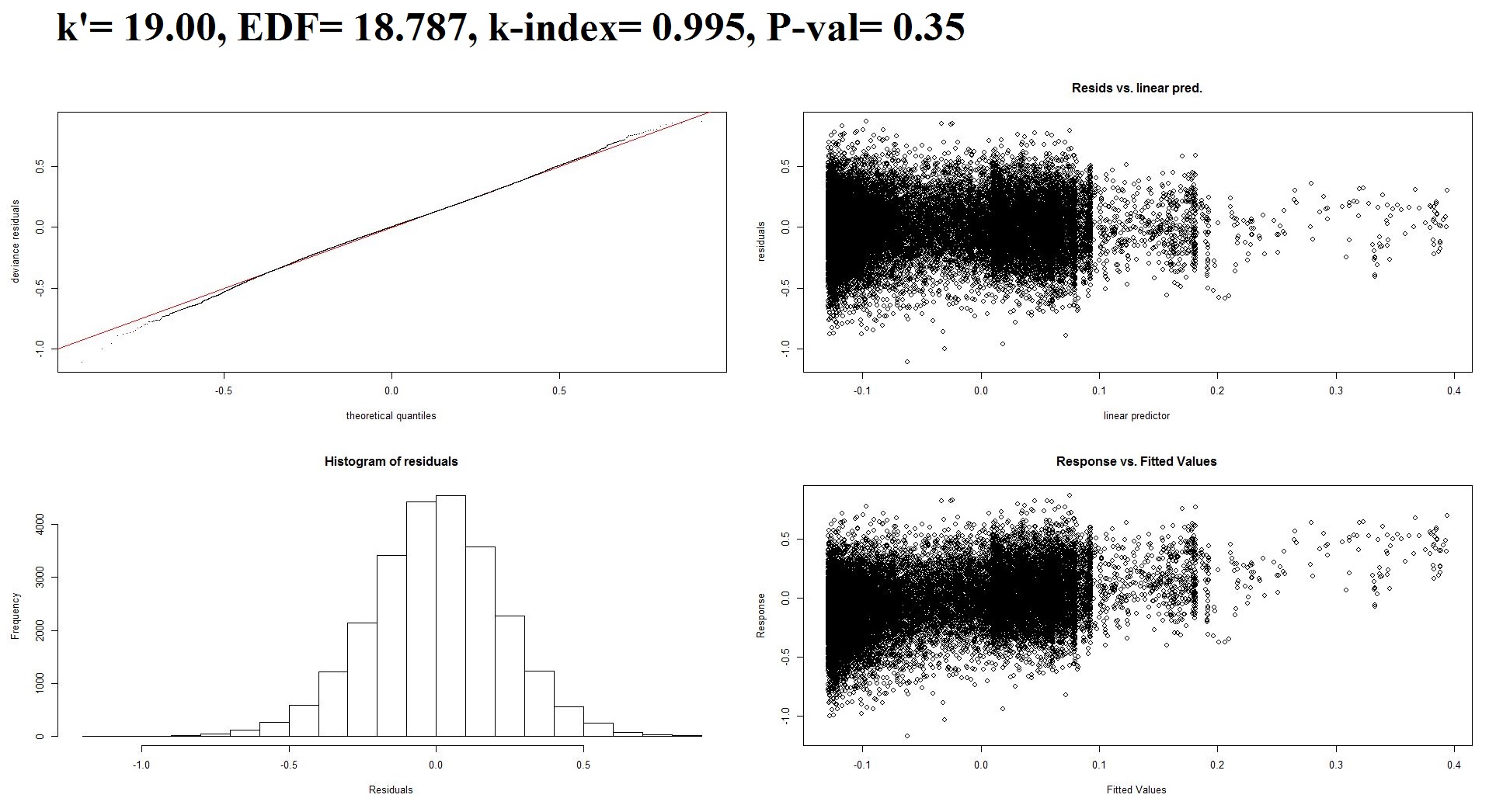

Supplement: Supplementary Figure 1 — Gene Ontology hierarchical diagram that shows significant enrichments of DCGs in lung adenocarcinoma and HNSCC colored in red (5% FDR). Scatter plots of expression vs. methylation in cancer and normal samples are reported for the top two genes (for reasons of brevity). [file Presentation1.ZIP › Supplementary Figure 8.JPG]

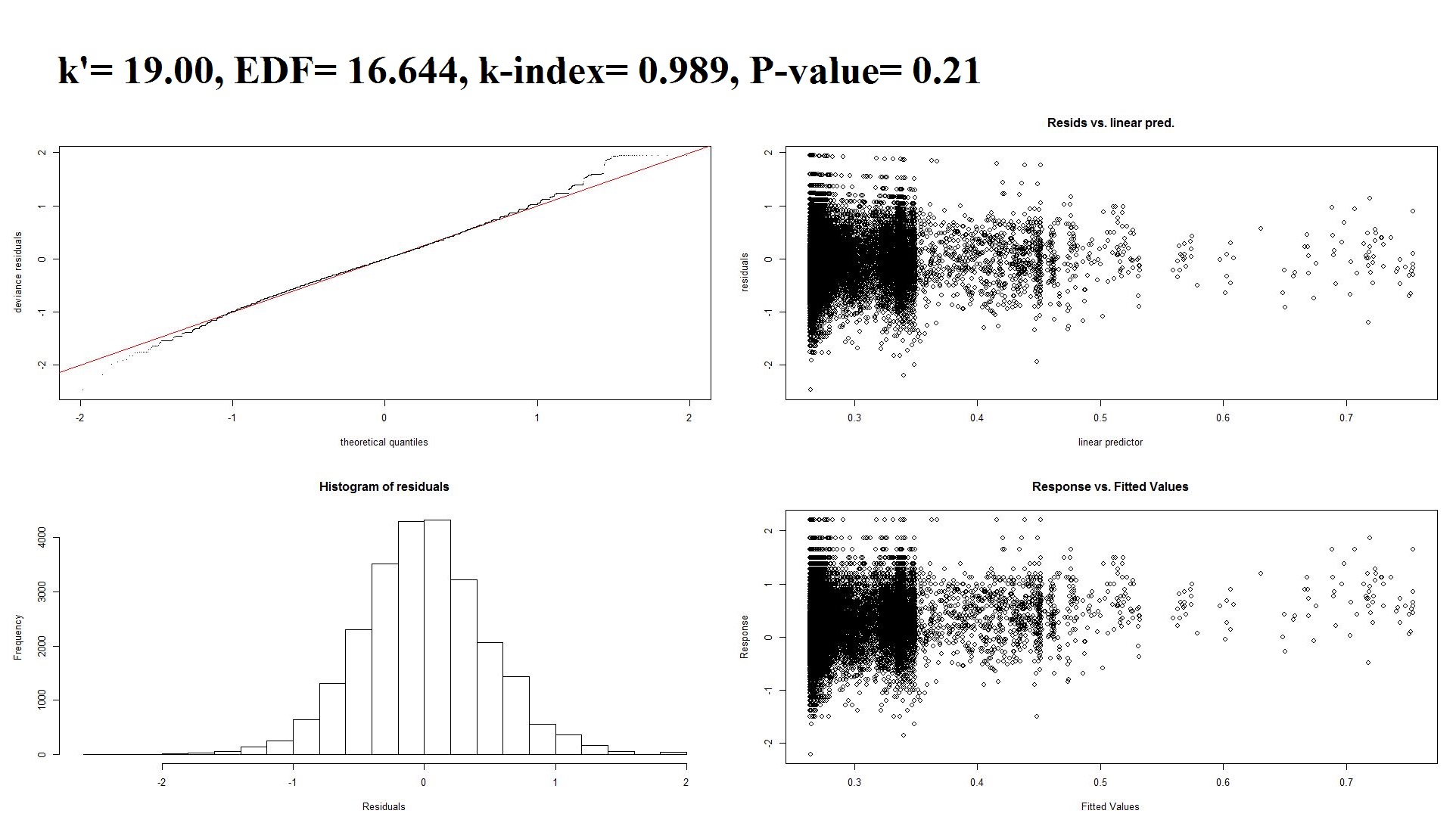

Supplement: Supplementary Figure 1 — Gene Ontology hierarchical diagram that shows significant enrichments of DCGs in lung adenocarcinoma and HNSCC colored in red (5% FDR). Scatter plots of expression vs. methylation in cancer and normal samples are reported for the top two genes (for reasons of brevity). [file Presentation1.ZIP › Supplementary Figure 9.JPG]

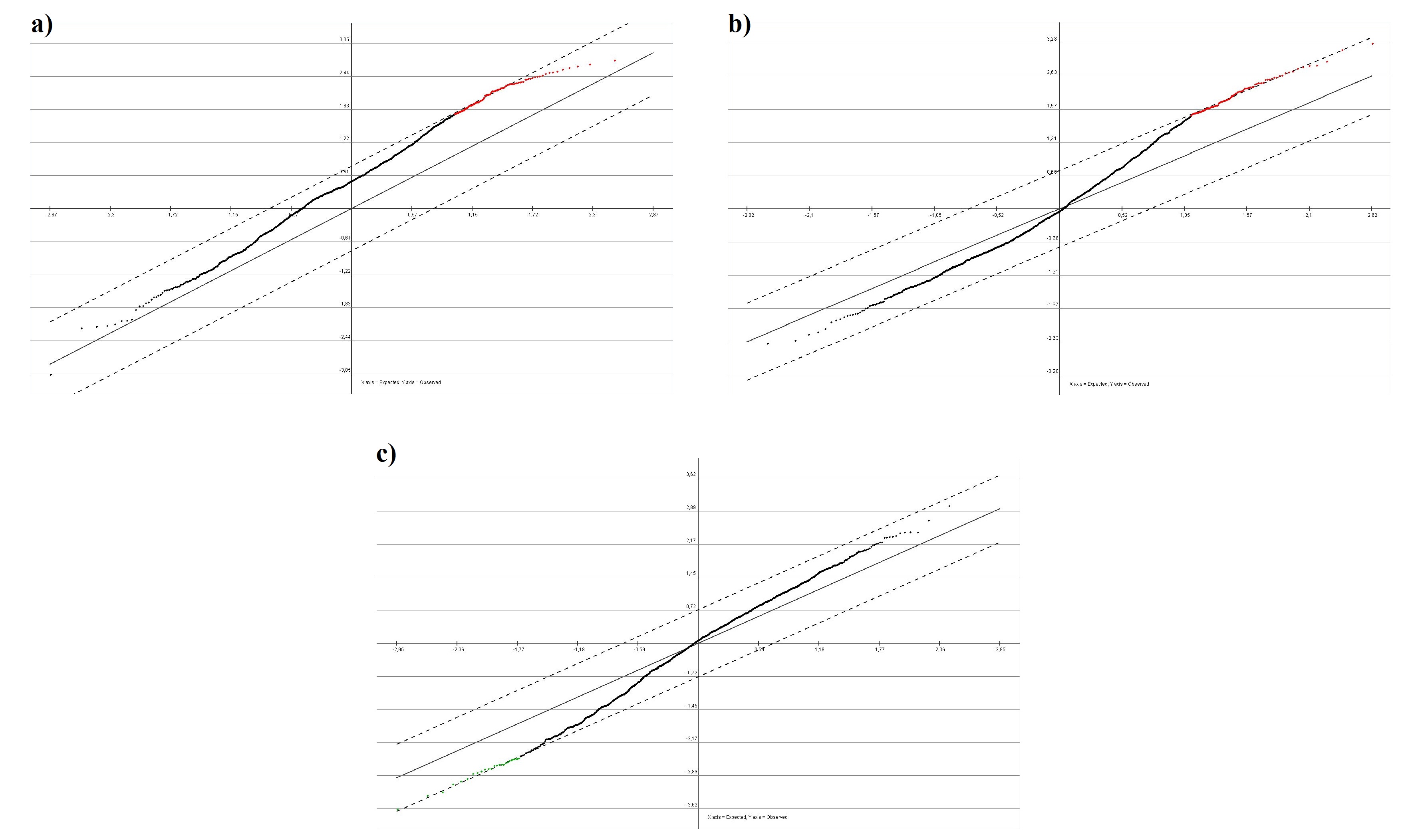

Supplement: Supplementary Figure 1 — Gene Ontology hierarchical diagram that shows significant enrichments of DCGs in lung adenocarcinoma and HNSCC colored in red (5% FDR). Scatter plots of expression vs. methylation in cancer and normal samples are reported for the top two genes (for reasons of brevity). [file Presentation1.ZIP › Supplementary Figure 10.jpg]

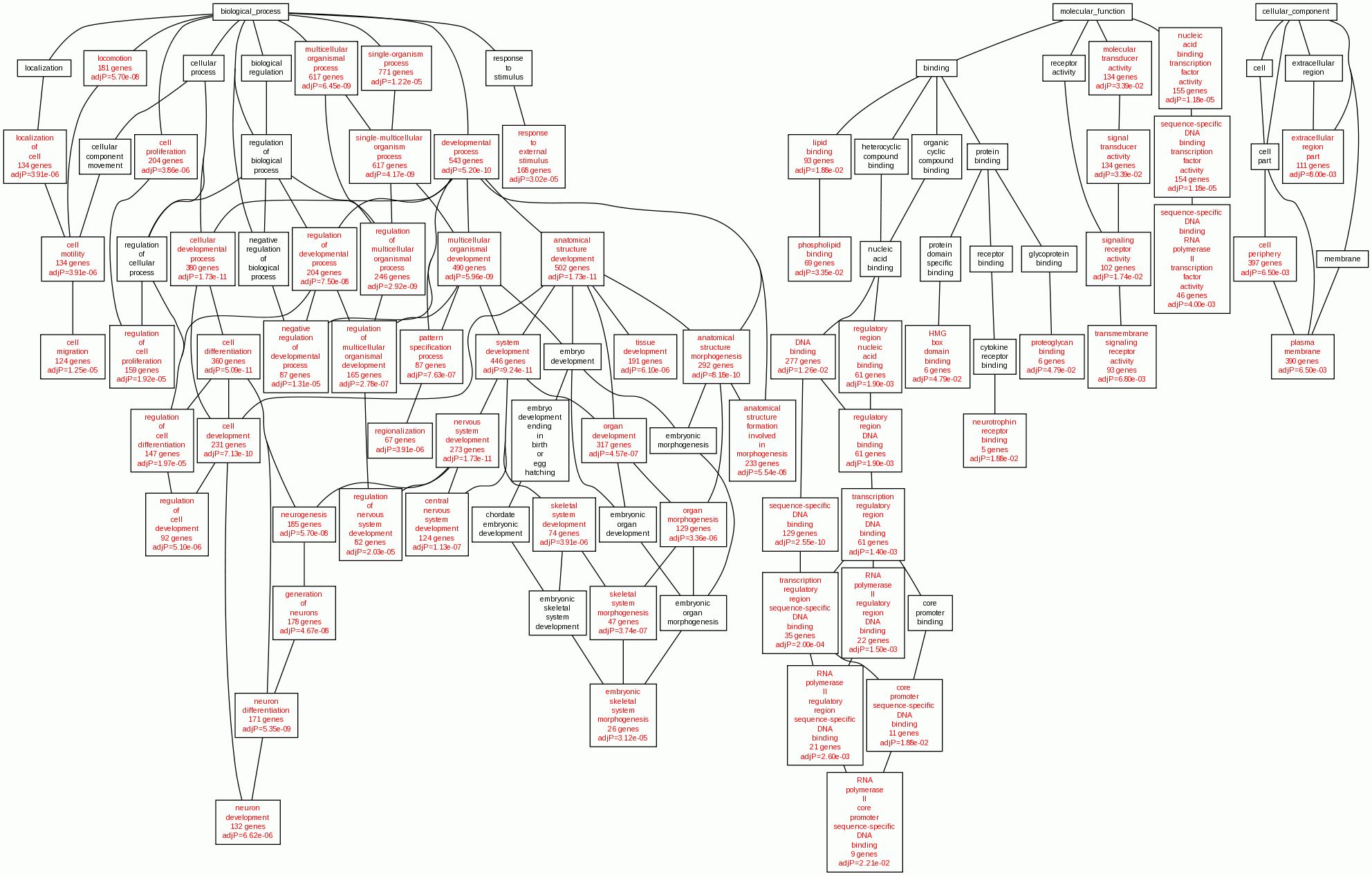

Supplement: Supplementary Figure 1 — Gene Ontology hierarchical diagram that shows significant enrichments of DCGs in lung adenocarcinoma and HNSCC colored in red (5% FDR). Scatter plots of expression vs. methylation in cancer and normal samples are reported for the top two genes (for reasons of brevity). [file Presentation1.ZIP › Supplementary Figure 11.jpg]
